# When do bursts matter in the primary motor cortex? Investigating changes in the intermittencies of beta rhythms associated with movement states

**DOI:** 10.1016/j.pneurobio.2022.102397

**Published:** 2022-12-21

**Authors:** Timothy O. West, Benoit Duchet, Simon F. Farmer, Karl J. Friston, Hayriye Cagnan

**Affiliations:** 1 Medical Research Council Brain Network Dynamics Unit, Nuffield Department of Clinical Neurosciences, University of Oxford, Mansfield Road, Oxford OX1 3TH, UK; 2 Wellcome Centre for Human Neuroimaging, Queen Square Institute of Neurology, University College London, London WC1N 3BG, UK; 3 Department of Neurology, National Hospital for Neurology & Neurosurgery, Queen Square, London WC1N 3BG, UK; 4 Department of Clinical and Movement Neurosciences, UCL Institute of Neurology, Queen Square, London WC1N 3BG, UK

**Keywords:** Neural activity, movement control, cortex, bursts, simulation, brain circuits, primary motor cortex

## Abstract

Brain activity exhibits significant temporal structure that is not well captured in the power spectrum. Recently, attention has shifted to characterising the properties of intermittencies in rhythmic neural activity (i.e. bursts), yet the mechanisms regulating them are unknown. Here, we present evidence from electrocorticography recordings made from the motor cortex to show that the statistics of bursts, such as duration or amplitude, in beta frequency (14-30 Hz) rhythms significantly aid the classification of motor states such as rest, movement preparation, execution, and imagery. These features reflect nonlinearities not detectable in the power spectrum, with states increasing in nonlinearity from movement execution to preparation to rest. Further, we show using a computational model of the cortical microcircuit, constrained to account for burst features, that modulations of laminar specific inhibitory interneurons are responsible for temporal organization of activity. Finally, we show that temporal characteristics of spontaneous activity can be used to infer the balance of cortical integration between incoming sensory information and endogenous activity. Critically, we contribute to the understanding of how transient brain rhythms may underwrite cortical processing, which in turn, could inform novel approaches for brain state classification, and modulation with novel brain-computer interfaces.

## 1 Introduction

Rhythmic activity from populations of neurons, as is routinely summarised using the power spectrum, is often leveraged to characterise neural activity from different brain regions (Keitel and Gross 2016; Mahjoory et al. 2020), behavioural states (Siegel et al. 2012), and pathologies (Brown et al. 2001; Schnitzler and Gross 2005). However, when analysed in time, neural rhythms often resolve into a succession of intermittent, transient events (Baker et al. 2014; van Ede et al. 2018; Feingold et al. 2015; Fingelkurts and Fingelkurts 2010; Freeman 2004; Friston 1997) that can appear as sustained oscillations when investigated using trial averaged analyses (van Ede et al. 2018; Jones 2016). To understand how alterations in power are underwritten by the temporal restructuring of neural rhythms, it is necessary to explicitly quantify the duration, amplitude, and rate of transient events (Heideman et al. 2020).

Temporal intermittencies in neural rhythms (i.e., “bursts”) are known to be important in behaviours such as sleep (Adamantidis et al. 2019) and working memory (Lundqvist et al. 2016). In the healthy motor system, changes in the temporal organization of beta frequency (14-30 Hz) activity can predict behaviour beyond that achieved when using just the amplitude of beta activity (Enz et al. 2021; Hannah et al. 2020; Shin et al. 2017; Wessel 2020). Further, beta burst dynamics appear to be significantly altered in Parkinsonism (Cagnan et al. 2019; Deffains et al. 2018; Tinkhauser et al. 2017b), where they form a major target for adaptive deep brain stimulation (Little et al. 2016; Tinkhauser et al. 2017a). Properties of transient activity can, in principle, improve the accuracy of brain state classification and thus have the potential to inform stimulation controllers that are adaptive to changes in behavioural context.

In the context of motor behaviour, preparation and execution have been described in terms of event-related synchronization and desynchronization in the beta frequency band (Pfurtscheller and Lopes da Silva 1999). Movement imagery has also been linked to event-related desynchronization albeit with less power decrease at beta frequencies than that seen during movement execution (Pfurtscheller and Neuper 1997). When beta frequency activity is temporally resolved, changes in the rate and timing of bursts are associated with movement preparation, planning, termination or cancellation (Diesburg et al. 2021; Feingold et al. 2015; Khanna and Carmena 2017; Little et al. 2019; Torrecillos et al. 2018; Tzagarakis et al. 2010; Wessel 2020). Additionally, beta bursts are associated with effects that persist beyond their termination (Khanna and Carmena 2017; Torrecillos et al. 2018). Recent evidence also suggests that bursts reflect a competition between endogenous processing and external sensory responses that bias perception in the cortex (Karvat et al. 2021).

Taken together, we hypothesize that: (1) the temporal properties of beta bursts are altered between different movement states such as rest, movement preparation, movement execution, and movement imagery; (2) these changes in temporal organization reflect altered responses of the motor cortex to stochastic inputs, that arise from a reconfiguration of the underlying microcircuit, and thus, (3) that expression of bursts reflect a rebalancing of how the cortex integrates spontaneous and exogenous inputs.

To date, the mechanisms underlying burst activity have been described using relatively simple models, such as an excitatory/inhibitory network of Wilson-Cowan populations (Duchet et al. 2021; Powanwe and Longtin 2019; Xing et al. 2012) that are motivated by pyramidal-interneuron models of beta generation (Jensen et al. 2005; Kopell et al. 2011). These studies indicate that burst statistics are determined by interactions between synaptic noise and the connectivity parameters of any given model. This suggests that models constrained using burst statistics can more accurately infer underlying connectivity across states. In more structurally fine-grained models, work has demonstrated the importance of laminar-specific corticothalamic inputs, which given the right timing can generate short, high amplitude beta events in the absence of endogenous neural activity (Sherman et al. 2016). Whilst these models have been useful in understanding how to either experimentally or therapeutically modulate the mechanisms that give rise to beta bursts, it is still not known how changes in burst statistics between brain states may be underwritten by alterations in laminar specific excitability, and how spontaneous rhythmic activity interacts with exogenous inputs to the cortex.

Here, we aim to establish how alterations in the cortical microcircuitry are manifest in the burst statistics of beta rhythms recorded from large scale neuronal activity. To this end, we use a library of publicly available electrocorticography (ECoG) data recorded from participants performing a range of motor tasks (Miller 2019). We first investigated how rhythmic burst features in these data may enhance the classification of different motor stages—such as movement preparation, execution, and imagery—by providing information beyond that available in the time-averaged spectra. Secondly, using computational models of the motor cortex microcircuit constrained to explain the both the power spectra and bursting properties of the ECoG data, we characterise how biophysical parameters may modulate bursting dynamics in different brain states. Finally, we use this model to test the hypothesis that changes in spontaneous cortical activity can reflect an altered balance of how the cortex integrates endogenous and exogenous inputs.

## 2 Methods

### 2.1 Electrocorticography and Experimental Recordings

All experimental data was taken from an openly available library (Miller 2019) and published for use without restriction (https://searchworks.stanford.edu/view/zk881ps0522). Recordings were made for anatomical mapping in patients with epilepsy at Harborview Hospital, Seattle, WA, USA. All patients provided informed written consent, under experimental protocols approved by the Institutional Review Board of the University of Washington (see supplementary information VII). Data were recorded at the bedside using Synamps2 amplifiers (Compumedics Neuroscan). Visual stimuli were presented using a monitor running BCI2000 stimulus and acquisition programs (Schalk et al. 2004). Electrocorticography (ECoG) was recorded using grids and/or strips of platinum subdural electrodes placed via craniotomy. Electrodes had a 4 mm diameter (2.3 mm exposed) with 1 cm interelectrode distance and were embedded in silastic. Electrical potentials were recorded at 1 KHz using a scalp/mastoid reference and ground. Hardware imposed a bandpass filter from 0.15 to 200 Hz. Locations of electrodes were confirmed using post-operative radiography. Exact details of the electrode localization methods can be found in Miller (2019).

**Figure 1:**
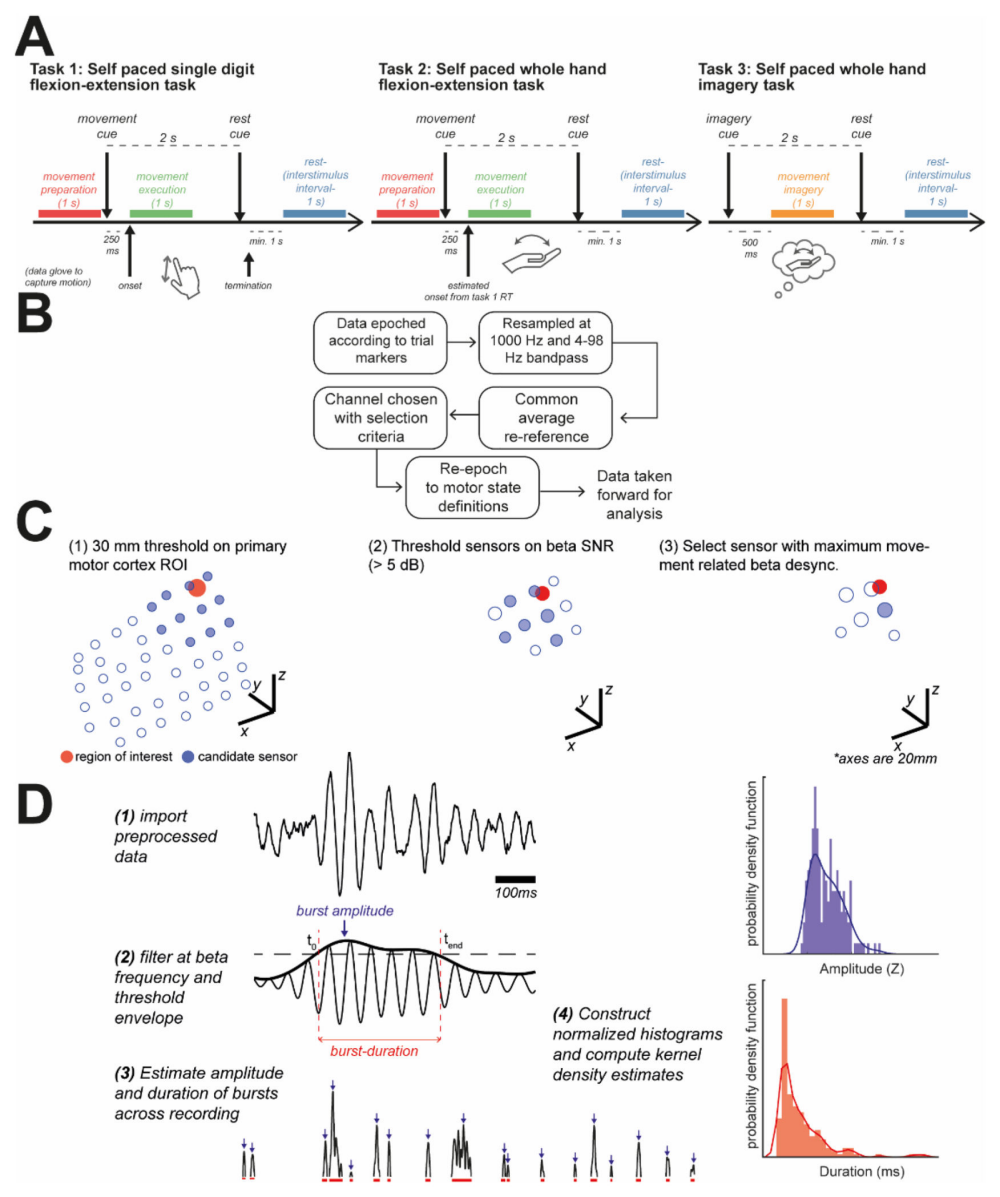
llustrated criteria for selection of ECoG channels and computed data features: spectra, and distributions of burst amplitudes and durations. **(A)** Data was taken from three motor tasks, requiring either self-paced flexion/extension of individual digits (task 1); or flexion/extension of whole hand (task 2); or imagery of whole hand movement (task 3). Data was epoched according to timings relative to those given in the figure. **(B)** Procedures for preprocessing data. **(C)** Illustration of channel selection procedure. Candidate ECoG channels (blue open circles) were selected (filled blue circles) using a 30 mm search radius of the ROI (MNI coordinate: [±37 -25 62]; red circle). All channels were thresholded at a +5 dB SNR threshold for the beta peak (see methods), finally channels were selected using the maximum movement related beta desynchronization. **(D)** Illustration of envelope threshold procedure to identify bursts. Samples of burst amplitudes and durations were used to construct histograms. The summaries of these distributions were then taken as the kernel estimate to the probability density function.

Data were taken from three different tasks as summarised below. For details of task structure and trial definitions please see figure 1A. Subject numbers represent the initial total available for each task, some subjects participated in more than one task. Data selection procedures are outlined in section 2.2.

*Dataset 1: Self-Paced Finger Movements (n = 9)* – originally reported in Miller et al. (2012). Participants were cued with a word displayed on a bedside monitor indicating which digit to perform a self-paced flexion and extension during a 2 s movement trial. Trials typically comprised 2-5 movements as recorded using a data glove. Movement blocks were interleaved with 2 s rest trials. Data were taken from the *“fingerflex”* folder of the Miller repository.

*Dataset 2: Basic Motor (n = 19) –* originally reported in Miller et al. (2007b) and Miller et al. (2010). Participants were asked to make either a simple repetitive flexion and extension of all the fingers, or a protrusion and retraction of the tongue at a self-paced rate (~2 Hz). Patients were cued with a picture of the body part to move, presented on a screen. Data were taken from the *“motor_basic”* and *“imagery_basic”* folders of the Miller repository.

*Dataset 3: Motor Imagery (n = 7) –* originally reported in Miller et al. (2010). Participants were asked to imagine making a simple repetitive flexion and extension of the fingers, or protrusion/protraction of the tongue at a self-paced rate (~2 Hz), matched to the task described for dataset 2. Imagery was intended to be kinaesthetic rather than visual- i.e., “imagine making the motion, not what it looked like”. Movement blocks lasted 2 or 3 s and were always followed by rest intervals of the same length. Data were taken from the “*imagery_basic*” folder of the Miller repository.

### 2.2 Pre-processing and Criteria for Data Selection

All ECoG recordings were processed as summarised in figure 1B. Large scale artefacts common across sensors were reduced by referencing electrodes to the common average. Channels with significant artefacts or epileptiform activity were visually rejected and excluded from the common average. Data were filtered between 4-98 Hz using a zero-phase (i.e., forward-backwards) FIR filter with -60 dB stopband attenuation. As the calculations of beta band signal-to-noise ratio (SNR) involved a comparison with the mean amplitude of the background, we chose a high-pass filter with a 4 Hz cut-off frequency to reduce the influence of 1/f increase in amplitude at low frequencies.

For each set of recordings, we selected one ECoG channel to carry forward for analysis. Data were selected to identify signals which were relevant to motor activity (i.e., spatially close to the primary motor cortex), of sufficient quality (i.e., good SNR of beta frequency activity), and functionally relevant (i.e., showing task related changes in synchrony). An illustration of the selection process can be seen in figure 1C. Channels were selected based on the following criteria: (1) select channels within 30mm of left or right primary motor cortex (MNI: [±37 -25 62]; Jha et al. (2015)); (2) threshold channels at +5 dB SNR for the beta band (14-30 Hz); (3) select a channel based on maximum SNR change between rest and movement/imagery. If no channels were found that matched these criteria the subject was removed from further analysis. The number of subjects whose data was carried forward for further analysis was: 5/9 subjects from dataset 1; 10/19 subjects from dataset 2; and 4/7 from dataset 3.

Only one channel per subject was selected for the full analysis. This is because the number of channels that passed the selection criteria was variable across subjects, and pooling between neighbouring sensors had the potential to bias the construction of burst distributions due to the superposition of multiple underlying cortical sources. This is supported by distributions of spectral and burst features computed in example subjects (supplementary methods VI; supplementary figure 7), that show that there is spatial spread of burst features across multiple sensors (Zich et al. 2020).

Data from each task were segmented into 1 second epochs. Details of epoching are illustrated in figure 1A. For dataset (1), kinematic data was available from a data glove worn during the experiment, and thus data was epoched according to movement onset (finger movements) determined using a threshold crossing on the smoothed movement traces. Data was segmented into *movement preparation* (-1250 ms to -250 ms relative to movement onset) and *movement execution* (0 ms to +1000 ms relative to movement onset) and then 1 s *interstimulus intervals* (ISI) blocks taken in between movement cues. ISI blocks were always at least 1 s away from a movement cue or movement termination. Throughout this paper we denote data from ISI blocks as “Rest”. Note that we left a 250 ms gap prior to movement onset to avoid non-stationarities that occur at the time that beta exhibits movement related desynchronization. This ensures that data arise from clearly defined states, rather than potentially containing data describing the transition between states. For datasets 2 and 3, movement kinematics were not available, and movement or imagination was cued by on-screen instructions. We therefore estimated movement onset using reaction times from dataset 1. If a subject also participated in dataset 1, we used their median subject-specific reaction time. For all other subjects, we used the group median. We took blocks of *movement execution* and *movement imagery* starting at cue onset plus this reaction time (lasting for 1 s in total). Movement preparation was defined as before.

### 2.3 Data Features: Spectra and Distributions of Burst Amplitude/Duration

Time series data were summarised using features derived from both spectra and bursts. We computed power spectral densities using Welch’s periodogram method with no overlap and a 1 s Hann window. Spectral features comprise the peak frequency within the beta band (14-30 Hz), wide-band SNR, and narrow-band SNR within the beta band (see supplementary methods I).

Bursts were defined using a threshold (75^th^ percentile) on the bandlimited envelope (Cagnan et al. 2019; Tinkhauser et al. 2017b). Note that thresholds were specific to each condition (i.e., over the concatenated epochs from data in each movement state). This was performed to avoid the bias towards burst effects reflecting simple differences in SNR that can occur with a common threshold (Schmidt et al. 2020). This does however mean that burst properties are relative to the condition specific signal power, with the threshold for burst identification in movement execution, being systematically lower than that for movement preparation due to the difference in beta power between these conditions.

For an illustration of burst definitions and the formation of summary statistics of burst properties, see figure 1D. Briefly, the distributions of burst amplitude or duration for each recording are summarised in terms of a probability density of arbitrary form (i.e., a kernel density estimate) which may be further reduced to its mean and standard deviation. Details of the procedure are given in supplementary methods III.

Overall, spectral features comprised: (1) wide-band SNR, (2) narrow-band SNR, and (3) peak frequency. Burst features comprised: (4) mean and (5) standard deviation of burst duration; (6) mean and (7) standard deviation of burst amplitude. Statistical tests were computed on log transformed data. For all features except peak frequency, a one-way ANOVA and post-hoc t-tests were used to test for changes in means of features between motor states. The distribution of peak frequencies was not found to be normal, therefore, a Kruskal-Wallis test plus post-hoc rank-sum tests were used to determine changes in mean.

Note that analyses of differences in signal recorded between motor states (results section 3.1) use the above quantitative *summaries* of the data features (i.e., the seven features described above), whereas the model fitting procedures (results section 3.3) utilizes the full *continuous* data features (i.e., spectral density, and probability densities for bursts) to constrain model parameters.

### 2.4 Assessing Feature Nonlinearity: Comparison with Linear Surrogate Data

To assess the extent to which statistics of burst features in cortical signals encode information beyond that contained in the power spectrum, we used a comparison to surrogate data (Theiler et al. 1992). Following work characterising the degree of nonlinearity in beta bursts (Duchet et al. 2021), we use Iterative Amplitude-Adjusted Fourier Transforms (IAAFT; Schreiber and Schmitz 1996). The IAAFT surrogate method improves upon the simpler technique of constructing randomized-phase Fourier surrogates, by not only ensuring the power spectrum is preserved, but also that the signal’s probability density is preserved. This ensures that the surrogate reproduces the linear features of the data whilst destroying potential nonlinearities in the original time series. To compare data with IAFFT surrogates, we constructed 25 surrogate time series for each data set, and then took the feature average, computed in the same way as for the reference (i.e., the empirical or simulated) signals. We then computed the goodness-of-fit in terms of the R^2^, with R^2^ ≪ 1 indicating significant deviation of a data feature from that expected in the equivalent linear process.

### 2.5 Classification of Functional States with a Support Vector Machine

To determine the ability of different data features to decode the functional state from neural activity, we employed a classification approach. Prior to classification, we applied Linear Discriminant Analysis (LDA) to the data to reduce the dimensionality of the feature space to two LDA components. We then used a multiclass support vector machine (SVM) using error-correcting output codes to combine binary classifiers into an ensemble and applied this to the LDA feature space. Learners were implemented in MATLAB using iteratively optimized hyperparameters, and a Gaussian kernel set. Model performance was evaluated using five-fold cross validation and the area under the curve (AUC) of receiver operating characteristics (ROCs) across the folds. Plots of SVM decision bounds were computed using posterior probabilities of model predictions applied in a grid search across the feature space. In effect, these measures of classification accuracy constitute an empirical estimate of model evidence or marginal likelihood, where the model in question maps from a functional motor state to various data features.

### 2.6 Identification and Fitting a Model of Motor Cortex Population Activity

We used a neural mass model of population activity in the motor cortex microcircuit (i.e., Bhatt et al. (2016)) that incorporates a middle layer reflecting the presence of layer 4 cells in primary motor cortex (Yamawaki et al. 2014). This neural (state space) model formulation follows from the Wilson-Cowan population firing rate model (Vogels et al. 2005; Wilson and Cowan 1972). This model was first employed to describe the behaviour of large scale, aggregate, neural population activity such as thalamocortical oscillations (Wilson and Cowan 1973) and since has been used extensively to explain cortical and subcortical rhythms (Deco et al. 2009; Lea-Carnall et al. 2016; Oswal et al. 2021; Pavlides et al. 2015; Powanwe and Longtin 2019; Yousif et al. 2017). As a prelude to the current modelling, we compared the evidence for convolution models — specifically, extended Jansen and Rit-like models (Jansen and Rit 1995) — against the simpler Wilson Cowan like model. The Bayesian model comparison is detailed in supplementary methods V and supplementary figure 4. Supplementary figure 4 shows that the Wilson-Cowan formulation better fits the burst features as indicated by the superior model evidence. Unlike the Wilson-Cowan model, we also found that the Jansen-Rit model was not able to capture altered burst properties, reflecting motor state dependent changes – a critical component of our study. For these reasons we used the Wilson-Cowan formulation in the remainder of this work.

The states of the Wilson-Cowan model depict *instantaneous* changes in the average firing rate of a population of neurons (spikes s^-1^). This population activity *A(t)* represents the *expectation* of the fraction of *N* neurons to be active within a short interval $\Delta t:A(t)=\frac{1}{\Delta t}\frac{n(t;t+\Delta t)}{N}$. This quantity is commonly estimated empirically from the peristimulus time histogram, in which the number of spikes per unit time (i.e., a rate) across a range of time bins is calculated. Peaks in the population activity reflect synchrony *en masse* in the neural ensemble, and thus periodic dynamics reflect phase-locked spike wave synchrony. In the neural mass formalism (in which the Wilson-Cowan equations are derived), a population is assumed to be made of a large number of identical, interconnected neurons that allows for equivalence between single neuron spiking and population spiking rates (Gerstner et al. 2014), and as such, the models are parametrized using single unit firing properties.

Using a coarse-grained, simpler model means that issues of interpretability can become more vexed. A key instance of this is the potential dissociation between neuronal firing – as measured in terms of single unit activity — and the density dynamics of populations – as measured by local field potentials and multiunit activity. In selecting a Wilson-Cowan formulation, we are committing to an interpretation of the model’s latent states in terms of a beta-phase locked neuronal firing (an interpretation that is supported by experimental evidence laid out in the discussion section).

Individual Wilson-Cowan equations were used to describe each population (e.g., cortical lamina and inhibitory interneurons). Intra- and inter- laminar projections were modelled using a delayed connectivity matrix reflecting the pattern of connectivity outlined in figure 4A. The model is driven using 1/f^α^ noise generated using a fractional Gaussian process (Dietrich and Newsam 1993), with *α* a free parameter to be fit. For a full description of the model equations please see the supplementary methods IV. The model comprises three pyramidal cell layers (superficial *SP*, middle *MP*, and deep *DP*) plus one population of inhibitory interneurons (II). Each cell layer receives a self-inhibitory connection reflecting local synaptic gain control. The output of the model is a weighted sum (i.e., a lead field) of the layer specific firing rates with 80% contribution from deep layers, and 10% each from superficial and middle layers.

Priors on model parameters dictating intrinsic dynamics (e.g., time constants, firing rate properties, etc.) were chosen using a combination of sources: (1) we preferentially used the Allen Brain Atlas data portal (https://celltypes.brain-map.org/) and retrieved properties derived from human cortical cells; (2) when parameters were not available in Allen Brain Atlas, we used the NeuroElectro database (https://neuroelectro.org/) as an alternative. For both databases, multiple estimates were available per parameter, and so we used the mean and standard deviation to specify the respective expectations and precisions on (Gaussian) prior densities. The parameter priors are outline in supplementary table I. Interlaminar connectivity was parameterized to match the same ratios of synaptic gains described in Bhatt et al. (2016). Prior covariances between parameters were assumed to be zero. See supplementary table I for specification of parameter priors.

Systems of stochastic-delay differential equations were solved numerically using a Euler-Maruyama integration scheme. For details of incorporation of finite transmission delays, and integration of the resulting system of stochastic-delay differential equations, see supplementary methods IV. To fit models, we used an implementation of the sequential Monte-Carlo Approximate Bayesian Computation algorithm (SMC-ABC; Toni et al. 2009; West et al. 2021). We take forward the maximum a posteriori (MAP) estimate (the collection of modes of the marginal posterior distributions) of each parameter for additional simulations.

Model fits were assessed by the data used to fit them: *type A* – using the power spectra only; and *type B* – using both power spectra and burst features (features described in section 2.4 “Data Features: Spectra and Distributions of Burst Amplitude/Duration”). We fit models to the group averaged data features and reduced the spectra to isolate peaks using a non-overlapping sum of Cauchy functions (see supplementary figure 3A and supplementary methods II). When fitting models across different motor states, movement preparation state was treated as a baseline, from which all other states were modulated. Movement preparation was chosen as the baseline as the group averaged spectra from this condition exhibited the strongest beta band power. The posteriors of the movement preparation state provided empirical priors for the remaining models (i.e., Rest, Movement Execution, and Movement Imagery) that describe deviations from this baseline state (i.e. the movement preparation state was fit first using all free parameters (i.e., time constants, synaptic gains, sigmoid characteristics, properties of intrinsic and observation noise)). Remaining motor states were fit using a restricted set of free parameters incorporating laminar specific time constants, synaptic gains, sigmoid characteristics, and the slope/gain of 1/f^α^ innovation noise. All models were fit to the group averaged data features for each state.

### 2.7 Finding Parameters Responsible for Shaping Bursts

The posterior parameter estimates—under models of the motor cortex—were examined to identify parameters responsible for shaping burst properties. To do this, we individually manipulated the synaptic gain and gain parameters for the laminar specific inputs (a total of 18 parameters) on a logarithmic scale from -2.5 to +2.5 (equivalent to approximately decreasing or increasing the strength 12 times) in 25 steps. Each model was simulated for 48 seconds, and the following properties were estimated: the peak frequency of the spectrum, percentage change in power (from base model), mean burst amplitude, mean burst duration. Parameters correlating with each feature were then identified by estimating the Spearman’s rank correlation coefficient with the average of each feature (i.e., the expected value of the kernel approximation to the probability density function). This constitutes a sensitivity or contribution analysis: in other words, it assesses the degree to which changing synaptic parameters generate discernible differences in the space of data features.

As features may not correlate across the whole connectivity range due to, for example, the existence of bifurcations in the model, we computed correlations within a restricted range. The optimal range was identified by computing the Spearman’s coefficient between the parameter and mean feature value across all possible ranges, with a minimum window of 1/3 of the whole range examined (i.e., 8 steps in connectivity strength). Correlations were thresholded using a Benjamini-Hochberg correction to set the False Discovery Rate to 10%, and the range yielding the largest coefficient was selected. The correlation between average burst duration and parameter scaling was used to choose the range, as this feature was found to have the largest association with interlaminar connectivity. Correlations with the other three signal features (peak frequency, mean burst amplitude and interval) were taken within this parameter range. Finally, candidate parameters were found by examining the correlation coefficients. To identify parameters engendering changes in burst properties—but showing minimal effects on spectra—we looked for those exhibiting clear correlations with burst features but not with spectral frequency/power.

### 2.8 Assessment of the Cortical Input/Output Fidelity and Relationship to Expression of Beta Bursts

We used the constrained models to understand how parameters responsible for modifying stochastic burst activity may regulate a trade-off between beta modulation reflecting spontaneous cortical activity versus that in response to exogenous input (e.g., as arising from sensory evoked potentials). To do this we delivered a train of inputs (modulations of asynchronous firing rate) to the middle pyramidal layer- the main recipient of thalamocortical afferents. We then assessed how this modulated beta bursts in deep cell layers – the predominant output layer of cortex (illustrated in figure 6). Inputs were given as a step function with bouts of length in seconds drawn randomly from a normal distribution with mean 500 ms and 150 ms standard deviation, and breaks drawn with mean 700 ms and 150 ms standard deviation. Inputs were multipliers on the stochastic firing rate and were set to 1x on the breaks and 3x (to test response to increase input rate) during bouts of upregulation. Fidelity of modulation was assessed by computing the Spearman’s correlation between the input (square wave of firing rate modulations) and output (square wave reflecting beta burst detection). We thus used this measure of input/output (I/O) fidelity to assess to what extent parameters known to regulate beta bursts also comodulate cortical integration of endo- and exo- genous activity.

## 3 Results

### 3.1 Properties of Beta Bursts in Motor Cortical Activity are Better than Spectral Features when Predicting Motor State

**Figure 2:**
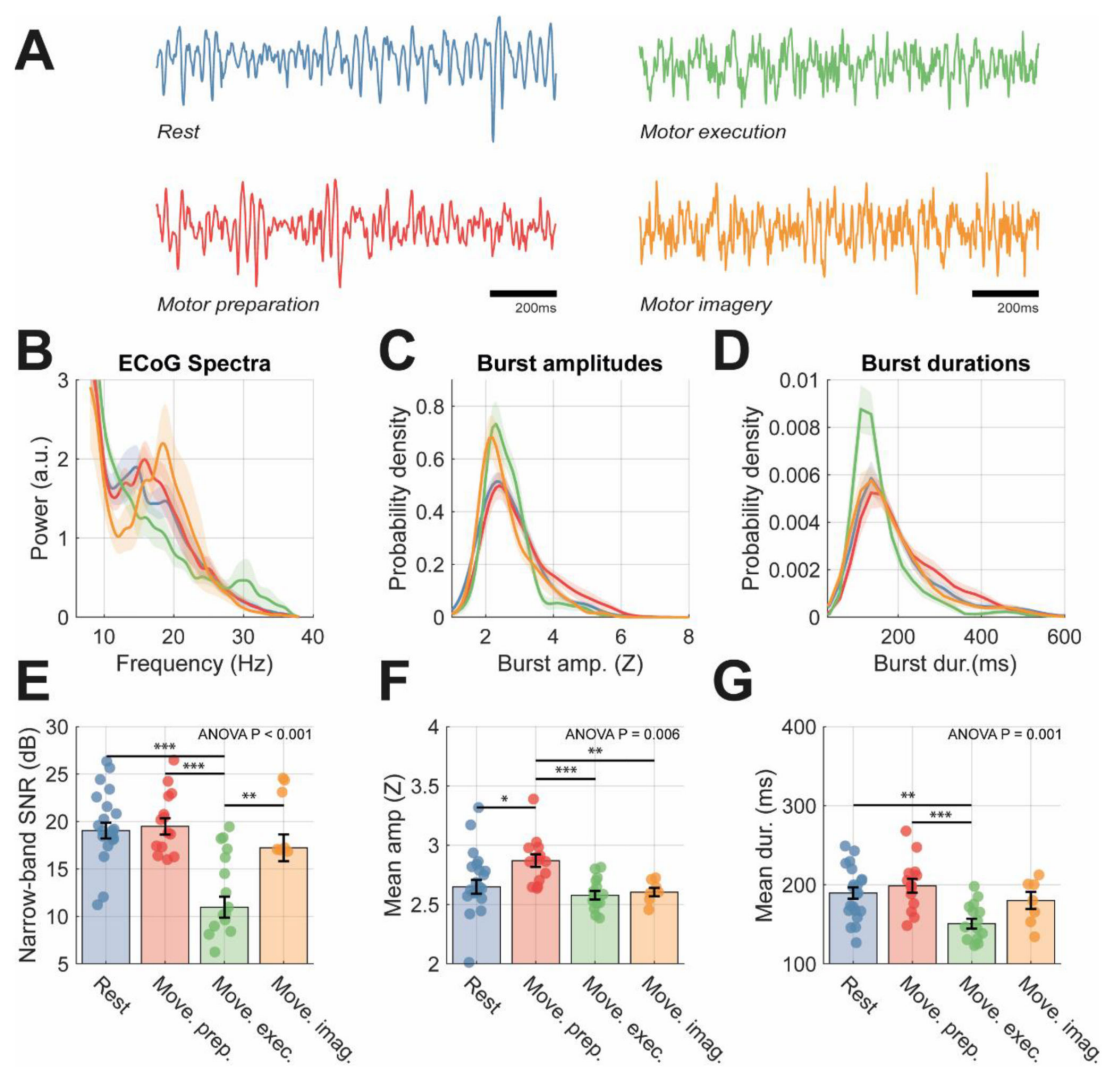
Analysis of recordings from selected ECoG sensors exhibit changes in properties of both spectral and burst features between motor states. Analyses were split among motor states: interstimulus interval (blue), movement preparation (red), movement execution (green), and motor imagery (orange). **(A)** Example 2 second time series of ECoG recordings for different motor states. Clear bursts of beta activity are apparent in rest, movement preparation, and imagery states. **(B)** Group average of normalized power spectra, **(C)** probability density of burst amplitudes (Z scores), and **(D)** probability density of burst durations (ms). Bar plots in (E-G) show data from individuals overlaid, with mean and standard distributions indicated by error bars. Data is shown for: **(E)** narrow-band SNR (dB); **(F)** mean burst duration (ms); **(G)** mean burst amplitude (Z score). Statistics indicate results of one-way ANOVA with bars indicating respective significant post-hoc t-tests between pairs of states. An analysis of the predictive value of burst vs spectral features in classifying motor states can be found in supplementary figure 2.

Data features summarising the spectra (e.g., peak frequency, power in band), and probability densities of bursting activity (e.g., mean burst duration/amplitude) were constructed from ECoG signals taken from the three datasets (see methods) and epoched to yield segments reflecting different motor states: rest (colour coded in blue throughout), movement preparation (red), movement execution (green), and movement imagery (orange). Data were selected from a sensor close to primary motor cortex that exhibited the largest movement related beta desynchronization (see methods section 1.2 for selection criteria). Example time series from the different motor states are shown in figure 2A which show clear bursts of 14-30 Hz beta activity in data from the different states. Spectra in figure 2B demonstrate movement related beta desynchronization in the group averaged spectra that is reflected in the change in 14-30 Hz narrow-band SNR from +18 dB to +11 dB from preparation to execution of movement (figure 2E; post-hoc t-test (40), P < 0.001). Changes were found in the wide band SNR (i.e., level of background noise indicating the overall signal quality) and corresponded to worsened recording quality during movement epochs (supplementary figure 1B). Beta desynchronization associated with movement is reflected also as a reduction in burst amplitudes (figure 2C and F; one-way ANOVA P = 0.006) and a shortening of beta burst durations (figure 2D and G; one-way ANOVA P = 0.001), although no significant changes were found in terms of the peak beta frequency or inter-burst intervals (supplementary figure 1 C and D, respectively). These results are robust to the choice of threshold used to define bursts, with peak discrimination between motor states occurring in the region of the 70^th^ to 85^th^ percentile (supplementary figure 5A and B). An analysis of the spatial distribution of ECoG activity over the cortex (supplementary figure 7) in a representative subject, showed that beta power, burst amplitude and durations peaked at a location close to the motor cortex.

To compare the predictive value of either spectral or burst features, we trained an ensemble of binary SVM classifiers to predict different motor states (supplementary figure 2). Decision boundaries (indicating > 50% or > 75% prediction success) between all four motor states were present for classification with burst features, and AUCs of the receiver operating characteristics (ROCs) showed good predictive value (AUC > 0.80). In contrast, classifiers using summary statistics derived from the power spectra could only separate features from movement preparation and execution states with AUCs > 0.5 (greater than chance level) and could not classify features derived from rest or imagery states. These results suggest that, when using band restricted information (i.e., within 14-30 Hz), the properties of bursting activity can significantly augment the prediction of motor states from brain activity.

### 3.2 Burst Features are not Predicted by Linear Models of the Data

**Figure 3:**
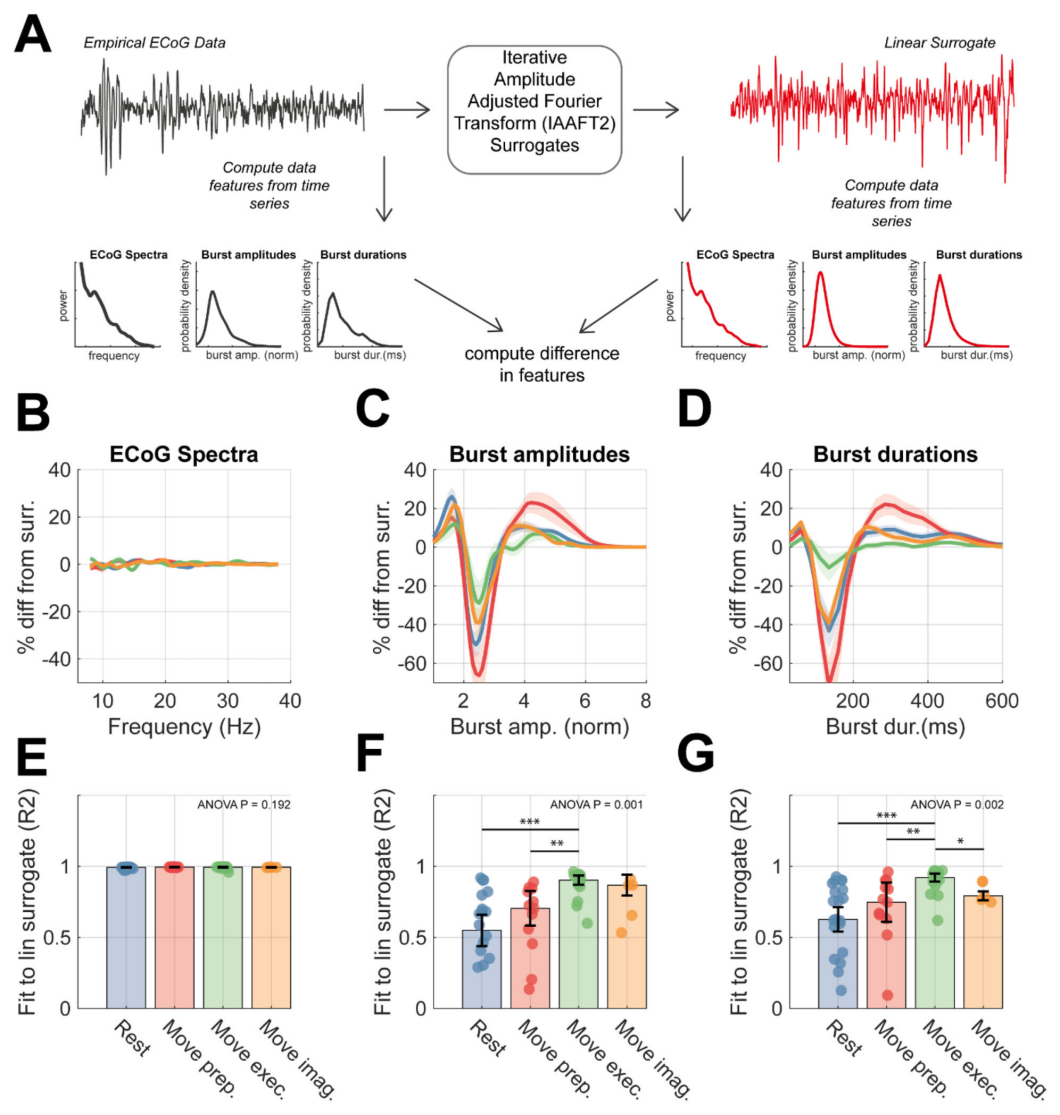
Comparison of empirical ECoG data with linear surrogates show that burst features represent significant signal nonlinearity that is modulated across conditions **(A)** The Iterative amplitude adjusted Fourier transform (IAFFT; see methods) was used to construct spectra-matched, linear surrogates (right) for each of the ECoG recordings (left). Spectral and burst features were computed for each signal, and the difference between the surrogate and empirical features were compared to assess the extent to which nonlinearities were present in data from the four motor states. **(B)** Plots showing the averaged difference between surrogate and empirical power spectra (computed as a percentage change). **(C)** Same as (B) but for distributions of burst amplitudes. **(D)** Same as (B) but for burst duration distributions. **(E)** Bar chart indicating the median goodness-of-fit of the surrogate to the empirical data feature with IQR shown by error bars. **(F)** Same as (E) but for burst amplitude distributions. **(G)** Same as (E) but for burst duration distributions. Statistics indicate results of one-way ANOVA with bars indicating respective significant post-hoc t-tests between pairs of states.

To further determine whether beta burst features reflect meaningful information about the underlying motor state, beyond that contained in the spectra, we compared empirical features with those computed from spectrally matched IAAFT surrogates (see methods section 3.4). In figure 3, we show a comparison between empirical data features and the average feature derived from surrogate data (n = 25) for each of the motor states. By design, the surrogates matched well to the power spectra of the data (figure 3B and E). Differences between the distributions of burst amplitudes and durations computed from the data or from linear surrogates (figure 3C/F and D/G, respectively) show that both features deviate significantly (median R^2^ < 0.80) from that expected under linear assumptions. Comparisons of the goodness of fits (R^2^) to linear surrogates showed that deviations in burst amplitude distributions from linearity were not identical across motor states (figure 3F, one-way ANOVA P = 0.002), with movement preparation and rest states showing reduced R^2^ values when compared to movement execution. Similarly, burst durations exhibited significant changes between states (figure 3G, one-way ANOVA P = 0.002) with data from the rest and movement preparation providing the greatest evidence for nonlinearity among all the motor states. These data suggest that burst features represent underlying nonlinearities in the data that are not captured in the power spectra alone. States associated with rest and movement preparation are associated with a higher degree of nonlinearity, especially when compared to movement execution. We next use a neural mass model to investigate the potential biophysical explanations for these differences.

### 3.3 Biophysical Models of Motor Cortex Fit Constrained to Fit Power Spectra do not Predict Distributions of Burst Features

**Figure 4:**
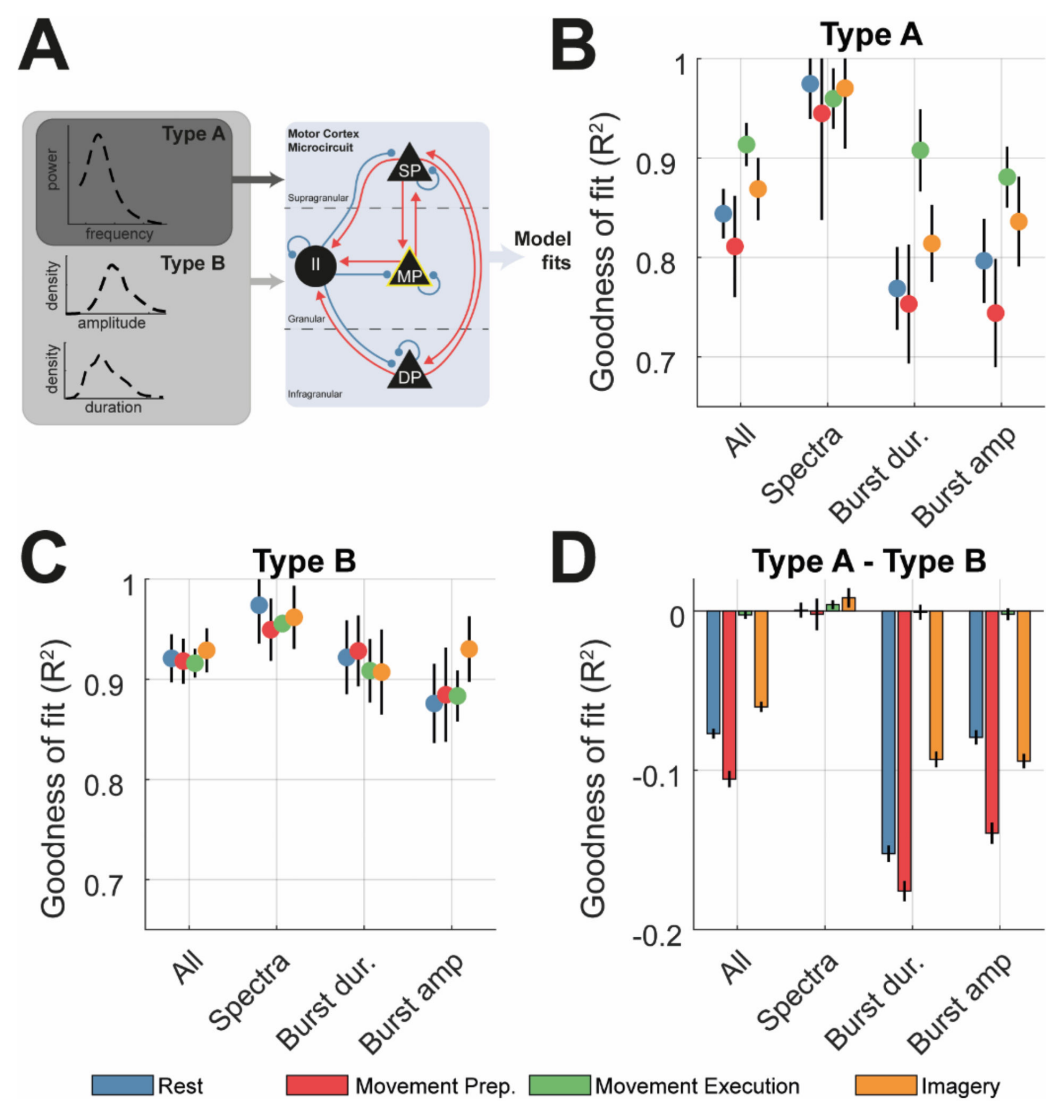
Comparison between type A (spectra only) and type B (spectra + burst features) fits of the motor cortex microcircuit demonstrates that spectral features are not sufficient to accurately constrain simulated burst parameters. Data features were constructed by simulating data using draws from the posterior distributions over parameters (n = 256). **(A)** Schematic of the motor cortex microcircuit model. Each black node represents a neural mass that is coupled with either excitatory (red) or inhibitory connections (blue). There are three pyramidal cell layers: superficial (SP), middle (MP), and deep (DP), plus an inhibitory interneuron (II) population. Model parameters were constrained using either pre-processed spectra (type A) or both spectra and burst features (type B) **(B)** Summary of the median ±SEM goodness of fit (R^2^) of the model to data from each state resulting from type A model fits. **(C)** Same as (A) but for type B model fits. **(D)** Difference in the goodness-of-fit (ΔR^2^) between type A and B fits. Negative values accuracy was greater in type B that type A fits.

The SMC-ABC algorithm was used to constrain a biophysical (neural mass) model of the primary motor cortex microcircuit to key data features (i.e., the power spectral densities and probability densities of burst duration/amplitude) from each of the four motor states. Models were fit to the group averaged data features and reduced spectra (see supplementary figure 3D-F and supplementary methods II). To assess the value of the power spectra in predicting burst features, fitting procedures were split into two groups depending upon the data features used: *type A* - constrained exclusively using the spectra, or *type B* – constrained using a combination of the spectra and distributions of burst amplitude and duration (figure 4A). Samples of the simulated time series using posterior estimates, as well as the fitted features are shown in supplementary figure 3.

*Type A* models fit well to spectra (figure 4B; all states R^2^ > 0.90) but showed that spectra were not sufficient to predict burst features accurately. Further analysis of the fitted features (supplementary figure 3E and F) showed that predicted distributions of burst amplitudes were attributable to smaller amplitude bursts than those observed in the experimental data, and burst durations were shorter than predicted in the case of rest and movement preparation (blue and red, respectively; R^2^ < 0.80). However, *type A* fits were sufficient to accurately recover the empirical distributions of burst durations in movement execution (figure 4C; green, R^2^ > 0.90).

In contrast, *type B* fits demonstrate that the model parameters could reproduce burst features (figure 4C), with a median fit of ~92% for all features. Complementary to the analyses of feature nonlinearity in figure 3, we show that the rest and movement preparation (the motor states exhibiting the highest degree of nonlinearity) gained the most (in terms of accurate predictions) from the explicit inclusion of burst features (difference of *type A* and *B* fits shown in figure 4D). In contrast, for data from movement imagery and execution, there was less gain in accuracy when explicitly incorporating burst features., we analysed the dependency of model inference upon the specific threshold used to defined bursts. This analysis shows that both parameters and models were not as well constrained at higher thresholds, due to the increase in variance in the summary statistics that arises from the drop in the number of instances identified.

The inadequacy of *type A* fits in predicting burst features (i.e., features withheld from *type A* model inversions) suggests that burst characteristics are the product of circuit mechanisms (and associated biophysical parameters) that are either independent or at most only weakly associated with those governing the power spectral amplitude and implies that features summarising temporal organization of bursts are important for informing neural models. Furthermore, burst features from periods of rest and preparation appear most different from those predicted using *type A* fits. In the next section we aim to identify parameters of the fitted microcircuit models of motor cortex underlying these changes in burst properties.

### 3.4 Modulation of Interneuron Activity in the Microcircuit is Predominantly Responsible for Modulation of Beta Bursts

**Figure 5:**
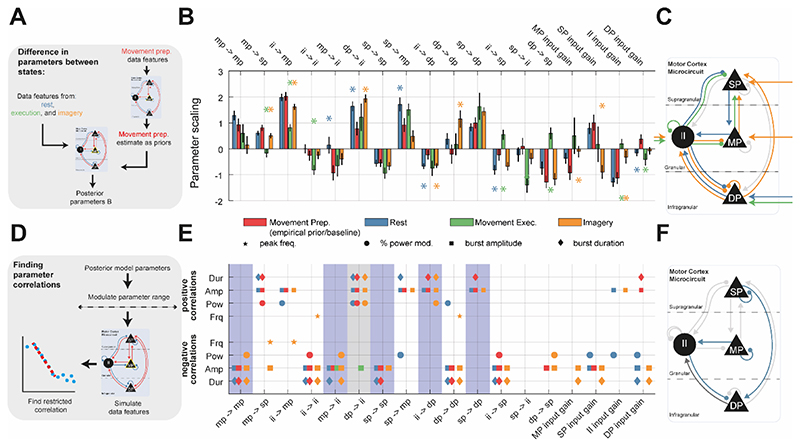
Results of the motor cortex model fits to ECoG data from motor tasks. Analysis shows posterior model estimates, as well as modulations in parameters from the baseline condition (movement preparation.), as well as correlation analysis of circuit parameters with the statistics of spectral and burst features resulting from posterior simulations. **(A)** Parameters of the model of motor cortex microcircuit were estimated from fits to group averaged data features from all four motor states using ABC-SMC. **(B)** Bar plot of the posterior model parameters with y-axis indicating log scaling ± the prior values at 0. Asterisks indicate statistically significant changes (Z-test of posteriors, P < 0.05) in parameters from the baseline state (movement preparation state; red). **(C)** Connections exhibiting a significant modulation are shown on the colour coded circuit diagram. **(D)** Modulations in parameters were estimated by first fitting to movement preparation data as a baseline state (using a wider set of free parameters, see methods), and then using these as empirical priors on the remaining models (using a smaller set of free parameters, see methods). **(E)** Parameters of the posterior models dictating interlaminar connectivity, and laminar specific inputs were then systematically examined for correlation with different data features. Correlations were performed on a restricted range with minimum range equal to 50% of the total parameter space tested(see methods). Parameter significance was determined using False Discovery Rate correction (10%). Grey bands highlight parameters that modulated both power and burst features. Parameters in light grey reflect those predominantly acting on burst features. **(F)** Connections and inputs exhibiting a significant correlation with either spectral and burst features (highlighted in grey) or exclusively burst features (blue) are shown on the colour coded circuit.

Parameters of the fitted models exhibited significant deviation from the empirical priors provided by model fit to movement preparation (i.e., the baseline state), and indicate changes in intra- and inter-laminar coupling (figure 5A and B). As expected, movement execution and imagery displayed the largest changes in parameters away from the movement preparation state. Movement execution largely involved changes in interneuron inhibition of middle and superficial layers (MP and SP, respectively; green figure 5C). Movement imagery and rest were associated with strengthening of reciprocal loops between deep and interneuron (DP and II, respectively) cell layers (orange and blue, figure 5C). An analysis of the laminar specific activity in the model (supplementary figure 8) demonstrated that these two layers (DP and II) were most active during burst activity in the movement preparation state, with firing rates in inhibitory layers greatest at the peak of a burst. We note that the baseline inhibitory tone (dashed line in supplementary figure) is high (~50 Hz) when compared to the activities of the pyramidal layers (~10 Hz). Both deep and inhibitory layers were heavily recruited during bursts and exhibit sustained rhythms outside of the main burst but with irregular phases that averages out across different instances.

To identify the parameters responsible for shaping beta burst features, we systematically altered interlaminar connection strengths and input gains, and then applied a restricted-window correlation analysis (see methods section 1.7) to detect co-modulation of certain parameters with the predicted spectral frequency, beta power, mean burst duration, or mean burst amplitude (figure 5D). Panels 5E and F show that several parameters affect these data features. For instance, the strength of DP→ II connectivity (highlighted in grey in figure 5E) positively correlates with both burst duration/amplitude and spectral power for 3 out of 4 of the states. Five parameters were found to predominantly modulate burst amplitude and durations independently of power (light blue in figure 5D, not showing correlation with spectral frequency or power for rest or movement prep.): MP → MP, MP → II, SP → SP, SP → DP, and II → DP. Notably, 4 out of 5 of these parameters involved modulation of inhibitory interneurons. To investigate how these parameters shape beta dynamics, we chose an example parameter—SP self-inhibition—that we took forward for further analysis. This was because: (A) it assumes a similar strength between motor states (figure 5B); and (B) it negatively correlates with both burst amplitude and duration but exhibits only limited effects on spectral peak frequency or power (figure 5E). It should be noted that this analysis depends on the minimum range for correlation detection, as well as the significance threshold. However, correlations with burst duration and amplitude were preserved despite these choices.

### 3.5 Increased Interneuron Inhibition of Superficial Layer Acts to Shorten Cortical Beta Bursts

**Figure 6:**
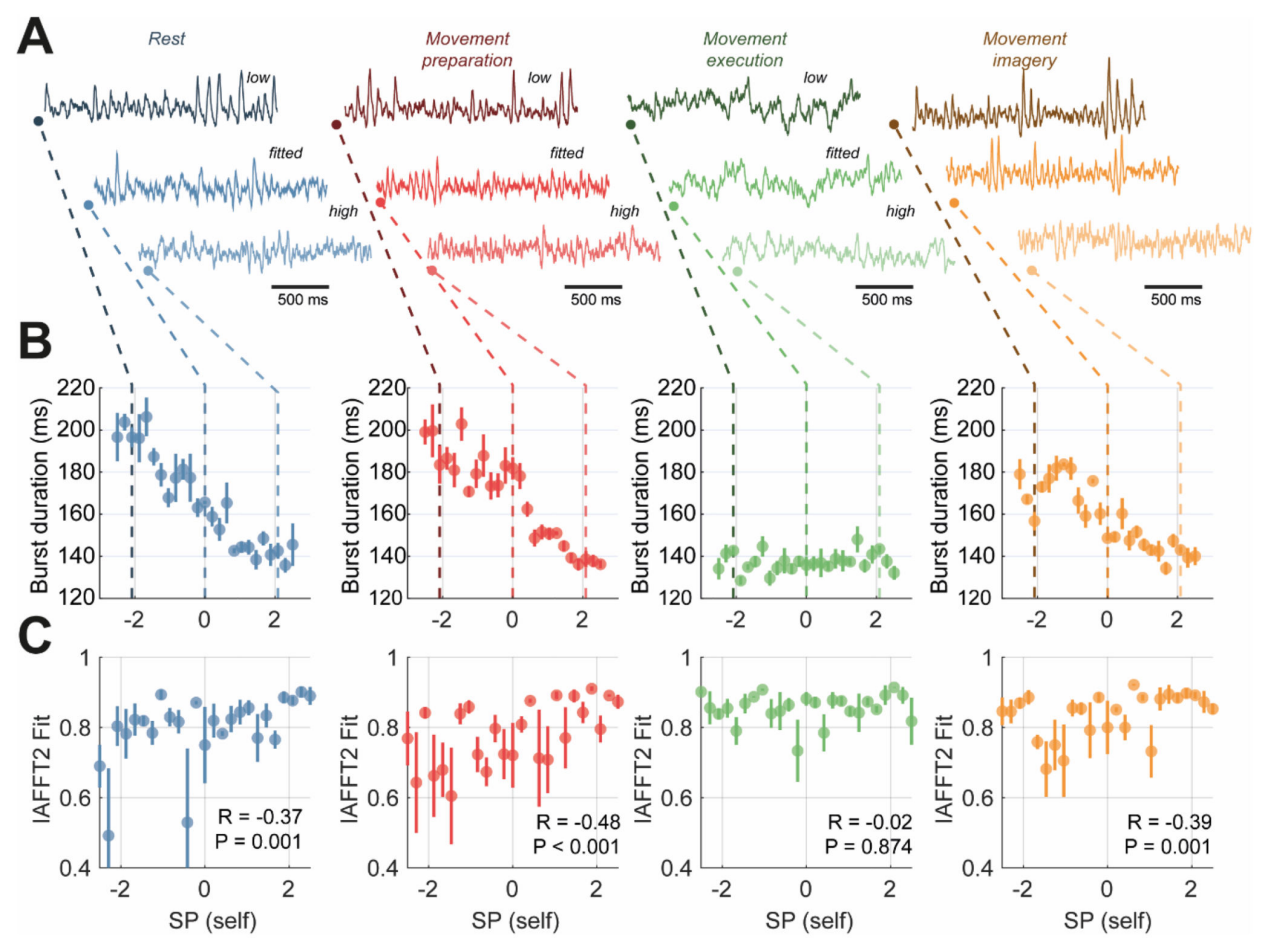
Detailed analysis of modulation of burst durations associated superficial pyramidal layer (SP) self-inhibition strength and corresponding correlations with signal features in terms of burst duration and nonlinearity. The level of superficial layer self-inhibition was taken forward as a control parameter following from the correlation analysis presented in figure 6F. Simulations were performed on a range of parameter values spanning -3 to +3 (log scaling from posterior). **(A)** 1.5 seconds of sample data simulated from each model of a motor state at either low (-2 scaling), fitted (0 scaling), or high (+2 scaling). **(B)** The mean burst duration is plot against the strength of SP cell input. All states excluding movement execution indicate existence of negative correlation between control parameter and burst duration. **(C)** The goodness of fit between burst duration distributions estimated from simulated data and linear surrogates (IAAFT processes) indicates that the degree of nonlinearity in the signals is anticorrelated to changes in the burst durations. Inset stats give the correlation coefficient and P-value for test of association between burst duration and the IAAFT R^2^ value.

We used SP self-inhibitory gain as a control parameter to investigate the effects of altered laminar specific inhibition on the temporal organization of simulated neural activity (figure 6A). Sustained oscillatory activity is observed in models fit to the data recorded during rest, movement preparation and movement imagery states, with strengthening of inputs of the superficial layer interneurons acting to extend bursts. Corresponding intermittencies in beta rhythms were graded, with burst durations shortening as SP self-inhibition was increased (figure 6C; blue, red, and yellow). In the model fit to data recorded during movement execution (in green), there was no periodic behaviour in the simulated traces generated by the model (figure 6A and B, green).

We also analysed changes in feature nonlinearity (using comparison to IAAFT surrogate method introduced in section 3.2; figure 6C), in which we found that IAAFT fits were negatively correlated with burst duration for three of the four states. However, the IAAFT R^2^ exhibited a higher degree of variance than burst durations, suggesting that the later might be a better proxy for system state in real-world data. These analyses demonstrates that the duration of temporal intermittencies of beta rhythms in the model, can be explained by the impact of biophysical parameters on the system, in this instance, SP self-inhibition acts to dampen rhythmic beta responses in motor cortex outputs.

### 3.6 The Temporal Organization of Spontaneous Beta Bursts Correlates with Cortical Integration of Exogenous Inputs

**Figure 7:**
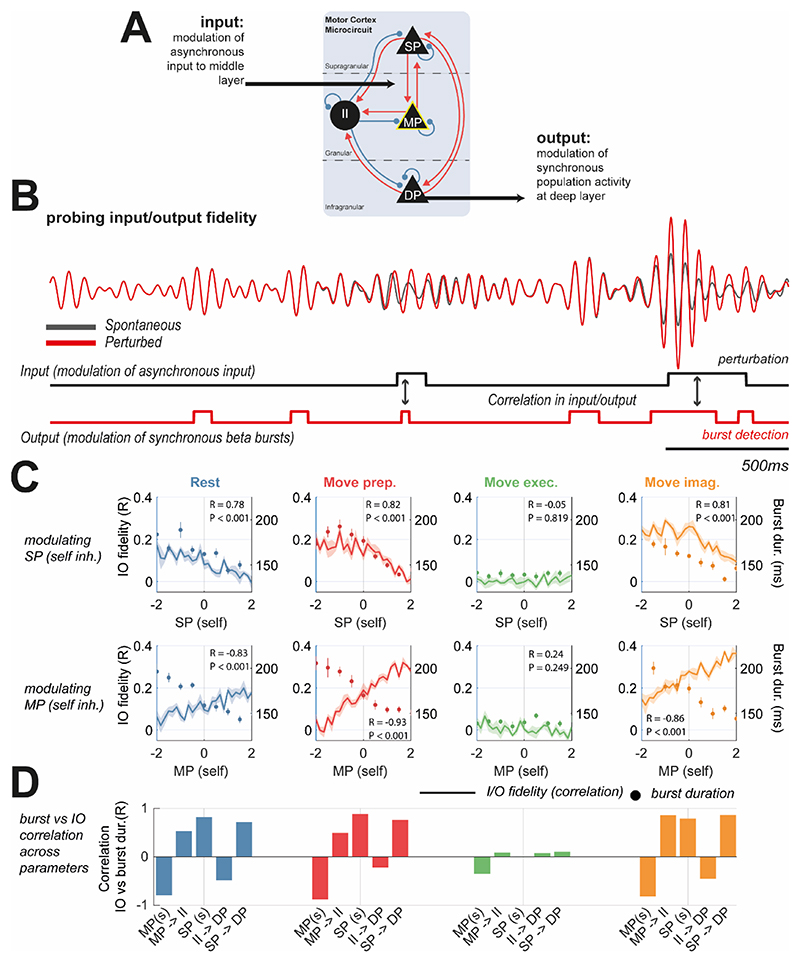
Parameters responsible for modulating burst properties do not uniformly alter the fidelity of synchronous cortical responses to exogenous inputs. **(A)** To probe the fidelity of cortical beta responses to changes in exogenous input, fitted models were used in an in-silico experiment. Asynchronous (stochastic) inputs to the middle layer were modulated with a square wave of random intervals. Beta burst detections in signals simulated in deep cell layers were taken as the outputs. The total “fidelity” of input/output (I/O) transmission was estimated using the rank correlation between these two square waves. **(B)** Example waveforms of the spontaneous (unperturbed; grey) activity, overlaid with perturbed (in red) activity matching the perturbation (i.e., modulation in noise to middle layer) seen below (black square wave). The output of the system matches the beta burst detections (red square wave) **(C)** Plots of I/O fidelity (left axis) versus burst duration (right axis) when modulating either SP or MP self-inhibition (top and bottom rows, respectively). Inset statistics indicate Pearson’s correlation R between I/O fidelity and burst duration and corresponding P value. **(D)** Bar plot indicating correlation between I/O fidelity and burst duration for five parameters known to modulate burst properties (see figure 5). Note direction of correlation changes dependent upon specific modulating parameter.

Lastly, we investigated the hypothesis that cortical beta burst properties reflect a trade-off between integration of spontaneous endogenous activity, versus that arising due to structured exogenous inputs (Karvat et al. 2021) (i.e., from sensory or higher order thalamus). In figure 7A and B we illustrate an in-silico experiment conducted on the models fit to different motor states in which we delivered patterned modulations of asynchronous (i.e., noisy) inputs to the middle layer of cortex (the main recipient of thalamic projections). We considered beta bursts in deep layer (the main projection layer of cortex) as the cortical output. We then measured the correlation between the input and output as an estimate of transmission fidelity.

The results in figure 7C show that whilst strengthening either SP or MP layer specific interneuron inhibition decreased the mean burst duration (right axes; shown by circle markers), the correlation with I/O fidelity was reversed dependent upon the parameter that was chosen for modulation. For instance, in the models fit to rest data, SP self-inhibition associated shortening of bursts correlated with a decrease in I/O fidelity. The opposite was true for modulations of MP self-inhibition. The analysis in figure 7D, shows that for all of the five parameters identified to modulate bursts (i.e., shaded blue in figure 5E), three parameters displayed positive correlations between I/O fidelity and burst duration.

An analysis of the excitation/inhibition (EI) ratio was performed by taking the ratio between the mean excitatory and inhibitory inputs to the DP cell layer (summarised in supplementary figure 6). We then investigated how EI changed between bursts. The results (supplementary figure 6) show that when modulating the strength of either SP or MP self-inhibition, both connections (as expected) tended to tip EI balance in favour of inhibition during beta bursts. We found that there was no consistent correlation with IO transmission fidelity, with positive or negative correlations found for modulations of either MP or SP inhibition, respectively.

These findings suggests that the properties of spontaneous cortical activity can reflect the underling balance of cortical integration of endogenous and exogenous inputs. This comes with the caveat, that to infer cortical processing from the temporal organization of spontaneous activity requires *a priori* knowledge of the laminar interactions that are responsible.

## 4 Discussion

### 4.1 Summary of Findings

Temporal dynamics of spontaneous activity in the brain contain significant information regarding how the cortex processes incoming information. Here, we have shown that motor states can be decoded from electrocorticography using features computed from narrow-band beta activity (figure 2). Our results show that these features aid classification (supplementary figure 2) and arise from signal nonlinearities that are not detectable in the power spectrum (figure 3). Further, evidence for nonlinearity was found to be greatest in data recorded during rest and movement preparation, indicating that the increase in information, beyond that available in the spectrum, and contained in the distributions of burst amplitude/duration, is highest in these states. Using a neural mass model, we then delved into the potential mechanisms and their functional significance. As expected, we found that neural mass models fit exclusively to spectra were not sufficient to accurately recapitulate the features of cortical beta bursts (figure 4). Analysis of the fitted model parameters between motor states found that burst properties are modulated predominantly by connections modulating interneuron inhibition and arise independently of connections modulating spectral amplitude or frequency (figure 5). Using the strength of superficial self-inhibition as an exemplar control parameter, we showed that layer specific inhibition acts to stabilize beta bursts in the time domain (figure 6). Finally, using the microcircuit model, we showed that the same parameters found to modulate temporal organization of spontaneous activity, also control the balance by which the cortex integrates exogenous inputs with that of ongoing endogenous activity (figure 7).

### 4.2 Intermittencies in Bursts can Discriminate Brain States Associated with Movement

Transient fluctuations in neural oscillations can contribute to the understanding of the organization of brain activity (Bonaiuto et al. 2021; van Ede et al. 2018; Feingold et al. 2015; Lundqvist et al. 2016; Sherman et al. 2016; Shin et al. 2017). Transients in beta oscillations, the focus of this study, are found in healthy sensorimotor cortex (Feingold et al. 2015; Hannah et al. 2020; Little et al. 2019; Rule et al. 2017; Wessel 2020), and also play a prominent raeiole in Parkinsonian electrophysiology (Cagnan et al. 2019; Tinkhauser et al. 2017b). Quantification of these intermittencies is beginning to build a taxonomy of bursts by identifying changes associated with different brain states and diseases (Deffains et al. 2018; Enz et al. 2021; Khawaldeh et al. 2020; Shin et al. 2017; Torrecillos et al. 2018). The discrimination of brain states by temporal features, as well as their transitory nature, makes them attractive targets for closed-loop approaches to neuromodulation, for instance using either beta (Little et al. 2016; Tinkhauser et al. 2017a), or theta and gamma (Kanta et al. 2019; Knudsen and Wallis 2020) based biomarkers.

The results reported here support this approach, by providing direct evidence that quantification of burst duration and amplitude, from narrow-band information can aid classification of motor states, in a way that is superior to that achieved when using spectral measures of beta power or peak frequency alone. Notably, we were able to discriminate between periods of rest and movement preparation, despite similar beta SNR observed across these states. These burst features are good candidates for control signals in closed loop neuromodulation, as they can be readily computed from narrowband data such as that available on current sensing/stimulation devices such as Percept (Medtronic) (Van Rheede et al. 2022) and they are known to be modulated by deep brain stimulation (Pauls et al. 2022). Additionally, motor state discrimination was enhanced compared to linear surrogates, with the degree of nonlinearity being largest during rest and movement preparation (figure 3). This technique has previously been deployed to show that Parkinsonian beta bursts are more nonlinear when compared to a medicated control state (Duchet et al. 2021). This suggests the possibility that biomarkers relating to signal nonlinearity can also form the basis for novel closed loop control algorithms (Jelfs et al. 2010).

### 4.3 Mechanisms and Functional Implications of Bursts in the Motor Cortex

If the statistics of bursts in rhythmic neural activity are discriminating features of brain states, then they may provide a window into the underlying changes in the generative neural circuitry. A prominent model of beta bursts, consisting of high amplitude, short duration events over the sensorimotor cortex, highlights the importance of synchronous subthreshold inputs to proximal and distal dendrites of pyramidal neurons (Bonaiuto et al. 2021; Sherman et al. 2016). Strong inputs to distal dendrites may then halt information processing by recruitment of inhibitory interneurons in the supragranular layers (Jones et al. 2009), that can lead to a reduction in pyramidal firing rates following cortical beta bursts (Karvat et al. 2021). As with any model, the conclusions drawn are a product of the data feature that they wish to explain. In the case of Sherman et al., (2016), bursts were comparatively rare (98^th^ percentile amplitude threshold; ~0.5 burst s^-1^), high amplitude events that permitted well stereotyped waveforms. In our own analysis, we aimed to explain more common events (75^th^ percentile amplitude threshold; ~1-1.5 burst s^-1^) that were typically multicycle. Similar events have been shown to exhibit burst distributions extending up to ~300ms in duration (Seedat et al. 2020). A focus on high amplitude beta events may occlude alternative mechanisms by which recurrent interlaminar interactions may either generate and/or sustain beta bursts lasting multiple cycles.

As changes in the temporal structure of beta rhythms between motor states are ascribable to alterations in inter and intra laminar connectivity, it follows that the amplitude modulation of beta oscillations may reflect changes in the cortical response to exogenous inputs. The cortex is known to exhibit context dependent changes in interlaminar propagation and laminar specific inputs (Kirchgessner et al. 2020; Takeuchi et al. 2011) yet limited information is known regarding the changes occurring during movement (Inagaki et al. 2022), and even less about how this relates to rhythmic neural activity. Our simulations demonstrate that input/output relationships between exogenous modulations in asynchronous firing rates and entrainment of cortical outputs at beta frequencies may change between brain states. Previous work has suggested that balanced excitation and inhibition can facilitate gating of neural signal propagation (Vogels and Abbott, 2009), we however did not observe a consistent relationship between EI balance and cortical beta responses to sensory inputs (supplementary figure 6), perhaps due to the high resting inhibitory tone in the model.

Further we show that properties of spontaneous activity such as burst duration can correlate with the fidelity of cortical integration of exogenous inputs (figure 7). However, the direction of this relationship is mechanism dependent – and thus inference of properties of cortical processing from analysis of spontaneous burst activity, would require a prior knowledge on the connections responsible for their modulation. Thus this work provides support for the idea that, given suitable generating circuitry, bursts in sensorimotor cortex can reflect a competition between spontaneous and sensory evoked activity (Karvat et al. 2021). It is possible that this cortical gating of sensory information may occur in tandem with transient changes to subcortical circuitry during sensorimotor processing (Mirzaei et al. 2017).

### 4.4 Model Inference and Intermittent Dynamics

This work also provides evidence that power spectra alone may contain insufficient information to accurately constrain parameters of nonlinear and/or stochastic models. Existing dynamic causal models of large scale temporal dynamics such as Parkinsonian beta bursts (Reis et al. 2019) or epileptic seizures (Rosch et al. 2018) appeal to fast-slow separation of time scales (i.e., the adiabatic approximation) in which changes in dynamics (i.e., bursting to quiescence) can be approximated by a model of fast (i.e., oscillatory) dynamics, with slow variables regulating the transition between states (Jafarian et al. 2021). In a similar vein, many phenomenological or statistical models describe bursts as a transition between discrete dynamical states (Heideman et al. 2020; Seedat et al. 2020). Other modelling approaches, such as that of Sherman et al. (2016), described above, take well constrained compartmental models that can describe high amplitude beta events, albeit with a specific pattern of input and in the absence of endogenous activity.

In this paper we take a different approach and treat bursts as the product of stochastic “quasi-cycles” that arise from noise driving a stable system such as a damped oscillator (Powanwe and Longtin 2019), that exhibit amplitude envelopes that can be modelled in terms of a drift-diffusion process (Duchet et al. 2021). Thus we use a model incorporating the full nonlinear transfer functions, and fit parameters of the resultant stochastic differential equations (West et al. 2021). Given the full breadth of information summarised by both the spectra and distributions of burst features, these models can well describe temporal dynamics of ECoG data in a parsimonious way without needing to appeal to modelling multiple states separately.

The distinction between generative models in which synaptic parameters fluctuate slowly and our model based upon stochastic dynamics speaks to an important distinction between explanations for itinerant dynamics of which beta bursts provide a good example. Technically, the first kind of generative model rests upon *structural instability*, where the itinerant changes in fast neuronal dynamics—and ensuing transients—are generated by changes in the fixed points of a system with the parameters of the equations of motion. In contrast, the second kind of generative model relies upon *dynamical instability*; namely, unstable (or weakly stable) fixed points to produce transient dynamics. This formal distinction has importance for understanding the biophysical mechanisms that generate bursts in population activity, as well informing stimulation approaches that aim to modulate them. For instance, in the case that bursts are the direct product of slow changes in neural circuits (i.e., invoking neural plasticity), then stimulation should directly target these mechanisms, whereas in terms of dynamical instability, stimulation can be patterned to with the aim of suppressing transient burst activity, or disrupting neural states that preclude them.

### 4.5 Limitations

A major problem when investigating changes in temporal dynamics between brain states arises from potential confounds that arise from the effects of changes in overall signal-to-noise of recordings. Although we found changes in the wide-band SNR (i.e., an estimate of signal quality between states (supplementary figure 1), alterations in burst amplitude did not correlate with either wide- or narrow-band SNR. Further, bursts were defined using a window-specific threshold, which prevents burst properties from predominantly reflecting SNR differences- a problem encountered when using a common (i.e., across condition) threshold (Schmidt et al. 2020). The robustness of using a fixed threshold of 75^th^ percentile is well supported following reports that specific threshold values do not qualitatively change outcomes of burst analyses (Lofredi et al. 2019; Tinkhauser et al. 2017b). Our analyses here support this and show that separability of motor states is maximal around the 70^th^ to 85^th^ percentile. Further, higher thresholds require more data to stabilize estimators of burst duration or amplitude. Accordingly, we found that at these higher thresholds, the precision of parameter inference from models was decreased.

We applied selection criteria (described in methods section 2.2) that lead to the rejection of ~40% of the available data, as in these subjects there was no beta peak at rest or movement preparation that was responsive to movement. Such stringent criteria were chosen to ensure that mechanistic modelling of the data was focused upon clear-cut cases in which intermittencies in beta were unobstructed by limits in signal quality. It is likely that in many cases of data rejection, the sparse spatial sampling of the ECoG grid may impede the recording from the underling cortical source.

Additionally, model inversion with Approximate Bayesian computation is susceptible to issues arising due to insufficiency of the summary statistics (i.e., the power spectrum, or distributions of burst duration/amplitude used here). More complete descriptions may be achievable with the bispectra (i.e., the Fourier transform of the third-order cumulant; Halliday et al. 1995). The results of the current study clearly call for development of generative models of these kinds of data features.

The ECoG signal arises primarily from an aggregate of currents flowing along the dendrites of spatially aligned pyramidal cells, a state not directly modelled by the Wilson-Cowan equations, which instead describe firing rates. Thus, the adoption of this model comes with an implicit appeal to an interpretation of the model’s states in terms of beta frequency phase locked neural firing. The frequency specific power of signals such as that measured in the local field potentials/ECoG and *single unit* firing rates can be dissociated (Confais et al. 2020; Rule et al. 2017). However, recent work by Karvat et al. (2021) has demonstrated that the instantaneous *population* firing rate (as commonly estimated in a peri-stimulus spike histogram, and the state described by the Wilson-Cowan model), exhibits correlation with high amplitude beta frequency bursts in the LFP. Previous work analysing spike/field synchrony has reported correlations spanning from weak (Rule et al. 2017), up to highly significant (Murthy and Fetz 1996a, 1996b; Peles et al. 2020).

The coarse graining of the neural mass model lumps diverse populations of interneurons into a single homogenous description. This limits the extent to which the model can explain important contributions from the neurochemical diversity of cortical interneurons (i.e., parvalbumin and somatostatin neurons). This diversity is known to play important roles in the modulation of large-scale rhythmic activity at both the gamma and beta frequencies (Chen et al. 2017; Lee et al. 2013; Veit et al. 2017). Future models can finesse the exploration of the role of interneuron diversity by inclusion of separate neural populations furnishing their specific electrophysiological properties and anatomical distributions.

### 4.6 Conclusions

This work provides significant evidence that the temporal properties of bursting intermittencies in brain rhythms contain unique information about the underlying circuits that generate them, beyond that more conventionally inferred from the power spectra of electrophysiological data. Furthermore, we have shown that burst features are nonlinear and are not simple predictions of the power spectra. Using a model of the primary motor cortex’s microcircuitry, we show that bursts can arise from stochastic dynamics, with properties that are predominantly modulated by laminar specific inhibitory loops. We have shown that this has important consequences for understanding information processing in cortical microcircuits, although simulations exhibit a non-trivial relationship between burst duration versus the responsivity of the cortex to exogenous inputs. These findings inform novel paradigms to understand the role of external perturbations such as electrical brain stimulation, in manipulating cortical computations when in the presence of spontaneous fluctuations in neural rhythms.

## Supplementary Material

Supplementary

## Data Availability

The data used in this study are freely available from a publicly available repository (https://searchworks.stanford.edu/view/zk881ps0522) described in (Miller 2019). The source code used to produce the results and analyses presented in this paper will be available as a Zenodo version-controlled repository accessible after publication of the final peer-reviewed publication of this manuscript.
