# Supplementary material for: When do bursts matter in the primary motor cortex? Investigating changes in the intermittencies of beta rhythms associated with movement states
Source: Prog Neurobiol. Author manuscript; Available in PMC 2023 May 9. (PMC7614511; doi:10.1016/j.pneurobio.2022.102397)
Supplement: Supplementary [file EMS175014-supplement-Supplementary.docx]

# Supplementary Information

## Supplementary Figures

### Supplementary Figure 1

| **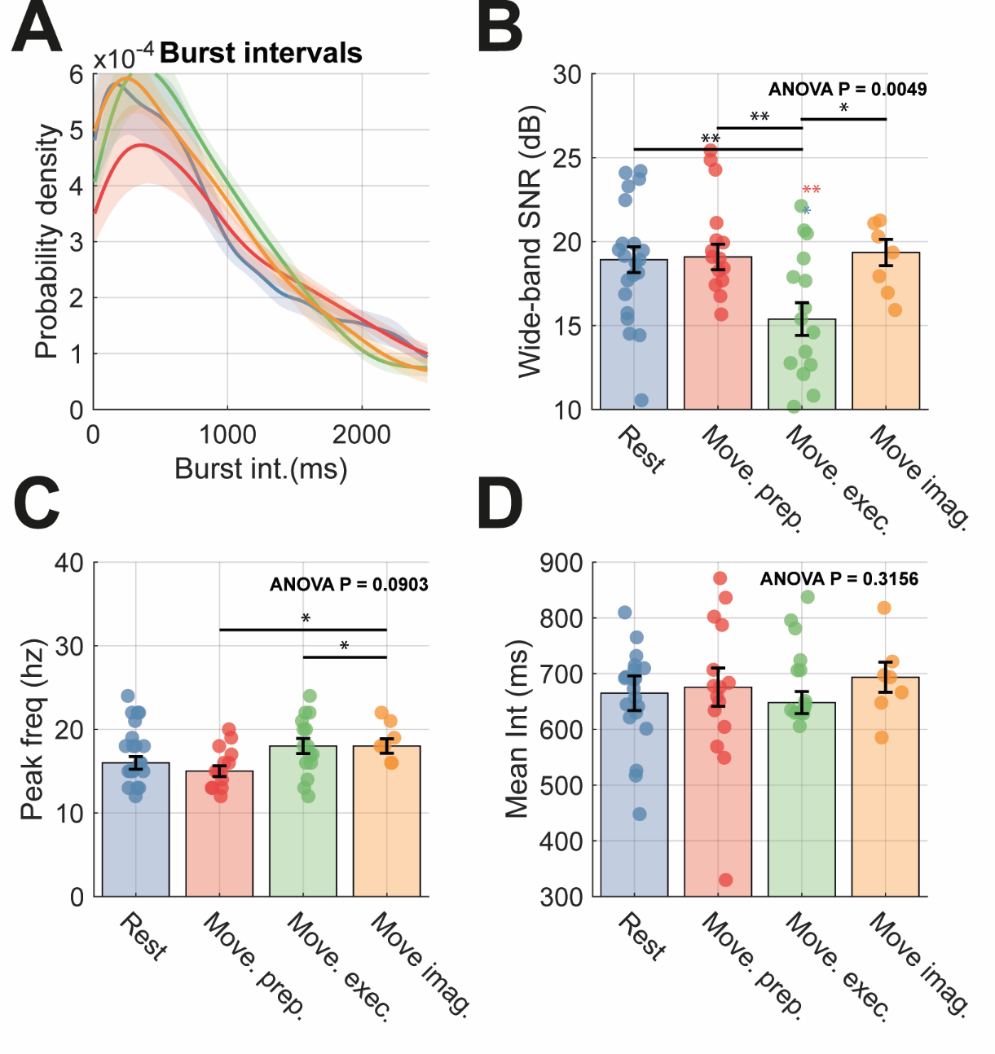** |
| --- |
| **Supplementary Figure 1 - Additional ECoG signal features compared between motor states. (A)** Probability densities of interburst intervals. **(B)** Bar chart to compare changes in the wide-band SNR of the selected ECoG channel. **(C)** Same as (B) but for peak beta frequency. **(D)** Same as (B) but for the mean interburst intervals. |

### Supplementary Figure 2

| **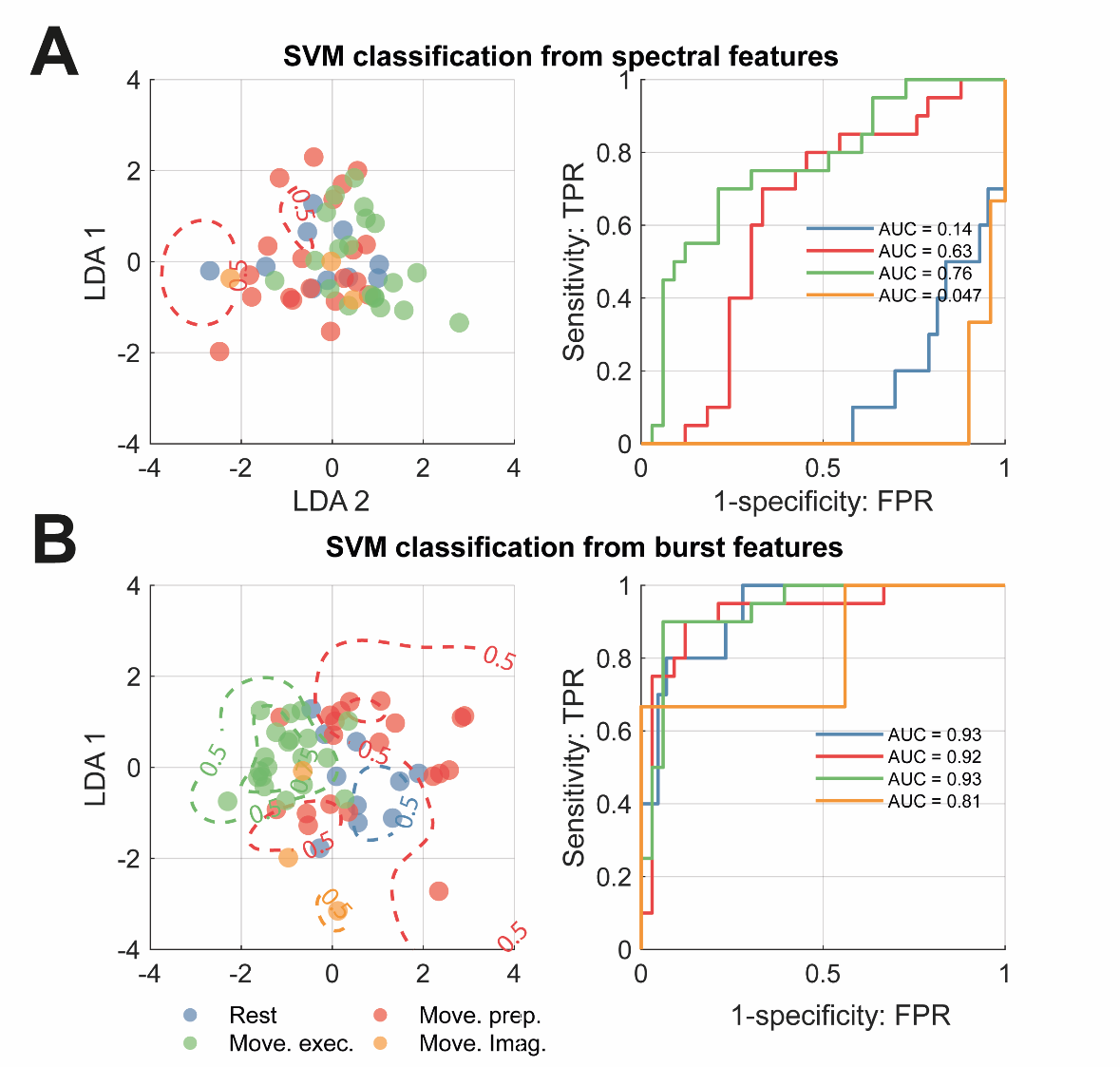** |
| --- |
| **Supplementary Figure 2 - Classification of movement states is superior when using beta burst features over that performed when using spectral features only. (A)** (left) Features of the ECoG power spectra (n=3) were projected onto a two-dimensional space using linear-discriminant analysis (LDA). Classification was then performed using ensembles of support vector machines on the first and second components of the LDA. The classification boundaries for each state are overlaid on the scatter plots of LDA features, at P = 0.5; and P = 0.75. (right) The receiver operating characteristics of each binary classifier are shown, with the area under the curve is inset. **(B)** Same as for (A) but when using burst features (n=6). |

### Supplementary Figure 3

| **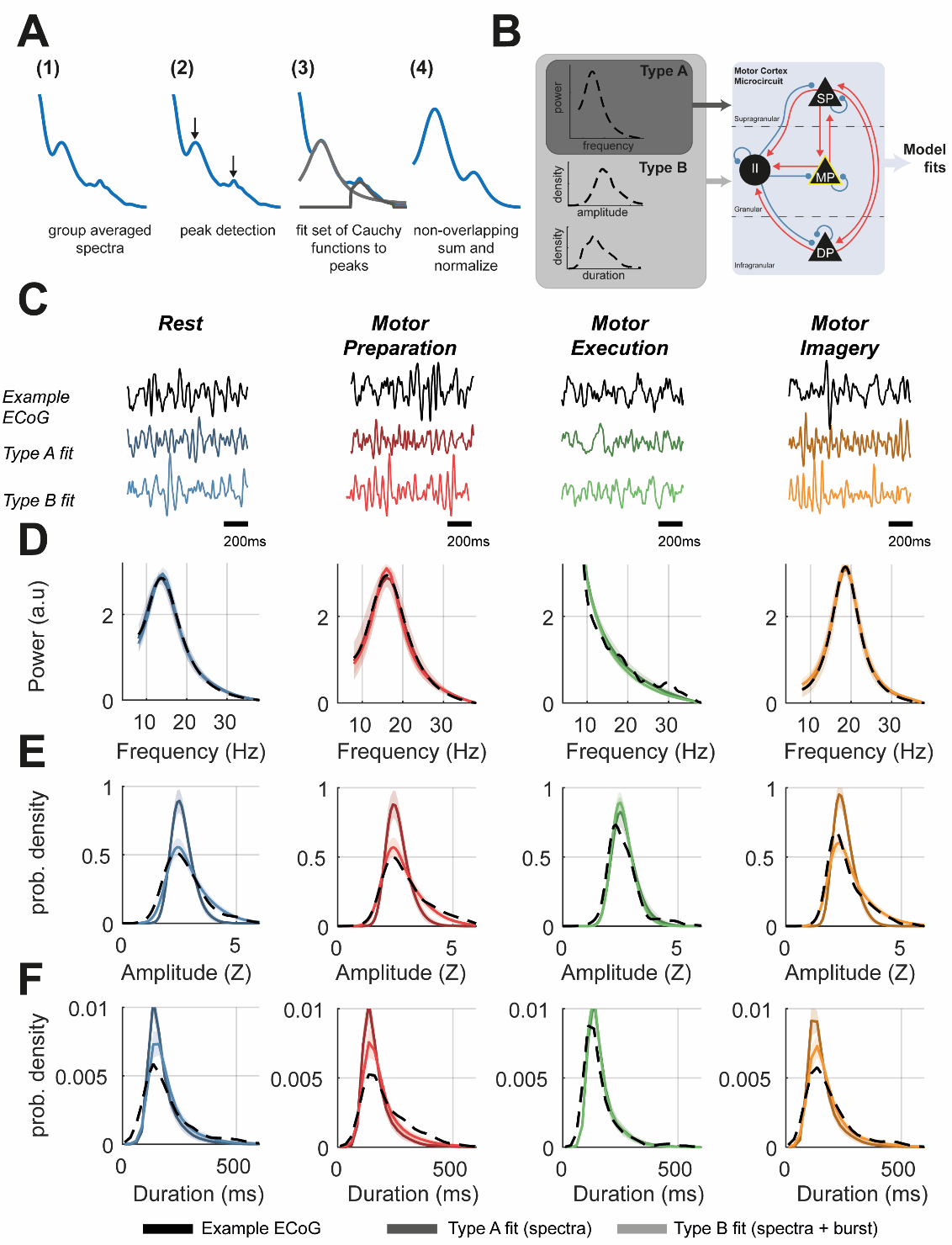** |
| --- |
| **Supplementary Figure 3 – Summary of model fits of motor microcircuit model to group averaged data features across motor states. (A)** Illustration of spectral preprocessing performed to isolate main peaks of spectra from 1/f background. **(B)** Schematic of the motor cortex microcircuit model. Each black node represents a neural mass that is coupled with either excitatory (red) or inhibitory connections (blue). There are three pyramidal cell layers: superficial (SP), middle (MP), and deep (DP), plus an inhibitory interneuron (II) population. Model parameters were constrained using either pre-processed spectra (type A) or both spectra and burst features (type B). **(C)** 1.5 second of example empirical data is shown from each motor state ( top; dark shade ), alongside those simulated from the posterior type A (middle; medium shade), or type B (bottom; light shade) fits. Data is shown from the interstimulus interval (blue), movement preparation (red), movement execution (green), and movement imagery (orange). Data features from the posterior model fits are shown for: **(D)** power spectra, **(E)** distributions of burst amplitudes, and **(F)** distributions of burst durations. |

### Supplementary Figure 4


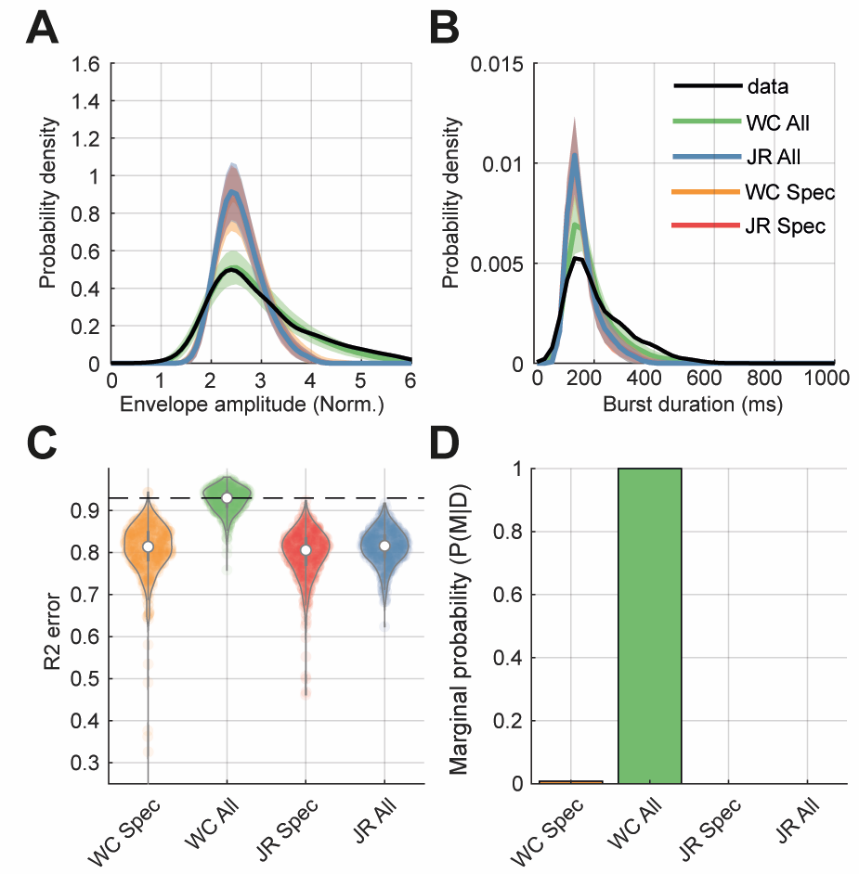


**Supplementary Figure 4 – Summary of Bayesian model comparison between two neural mass models to describe motor cortical microcircuitry: Wilson-Cowan (WC), or Jansen-Rit (JR) equations.** Models were fit using the SMC-ABC neural modelling toolbox and fit to either spectra exclusively (Spec) or a combination of spectra and burst features (All) from movement preparation. **(A)** ABC fits to burst amplitude distributions. **(B)**  ABC fits to burst duration distributions. **(C)** Violin plots indicating the distributions of pooled R^2^ error function for fits to spectra, and distributions of burst amplitude/duration. The median error (dashed line) was used as threshold by which to compute marginal probabilities. **(D)** The estimated marginal probabilities (P(M|D)) for each of the models.

### Supplementary Figure 5


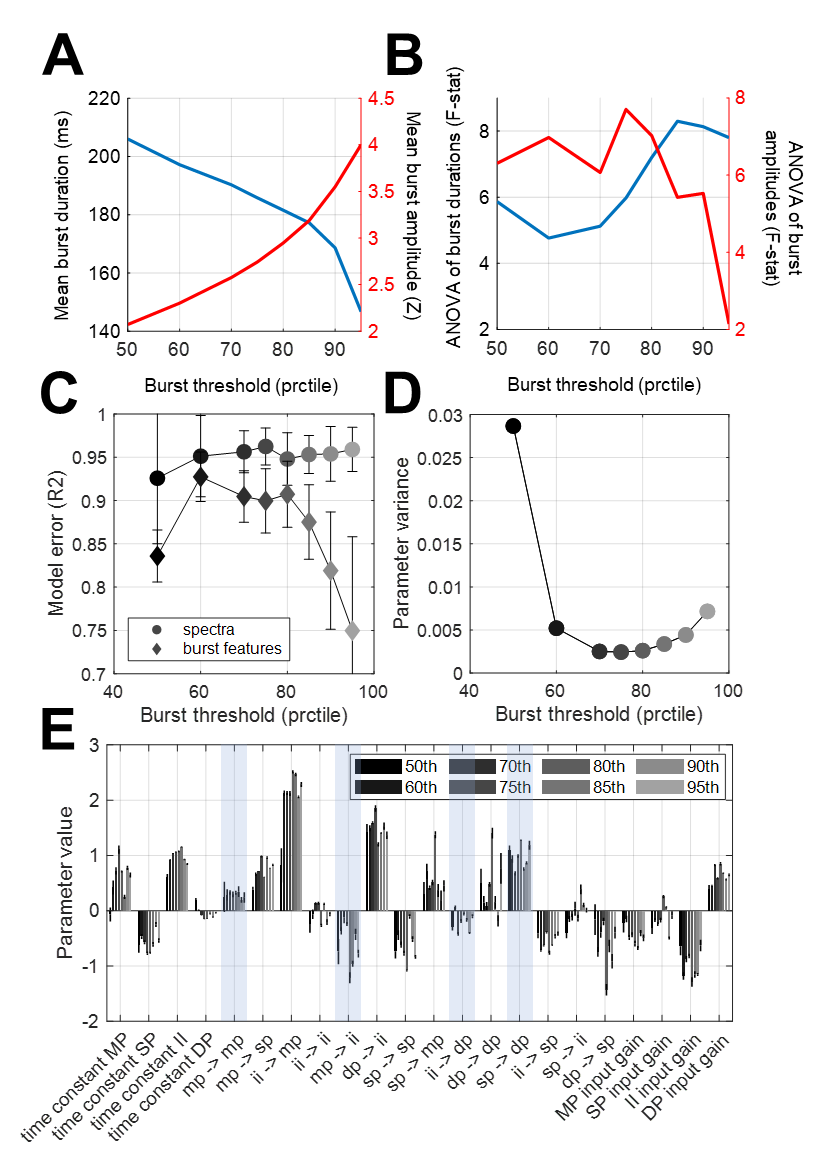


**Supplementary Figure 5 – Analysis of effects of threshold used to define beta bursts upon the accuracy of model fits and inferred parameters.** **(A)** Plot of mean burst duration (blue, left) and amplitude (red, right) as a function of burst threshold. Dashed lines provide comparison with a threshold free method for burst definition (a hidden Markov model; Quinn et al., 2019). **(B)** The resulting F-statistic associated with an ANOVA across motor states for burst durations (blue, left) and amplitude (red, right) as a function of burst threshold; SMC-ABC was used to fit models to data from movement preparation, using a range of burst thresholds. **(C)** Scatter plot of the accuracies of model fits to spectral (indicated by circles) and burst (diamonds) features. **(D)** Scatter plot of the average variances of inferred parameters as a function of burst threshold. **(E)** Bar plot of maximum a posteriori parameters from models fit across the range of thresholds. Error bars indicate the S.E.M. Blue highlights indicate parameters that were identified in figure 5 to be important modulators of beta burst activity.

### Supplementary Figure 6


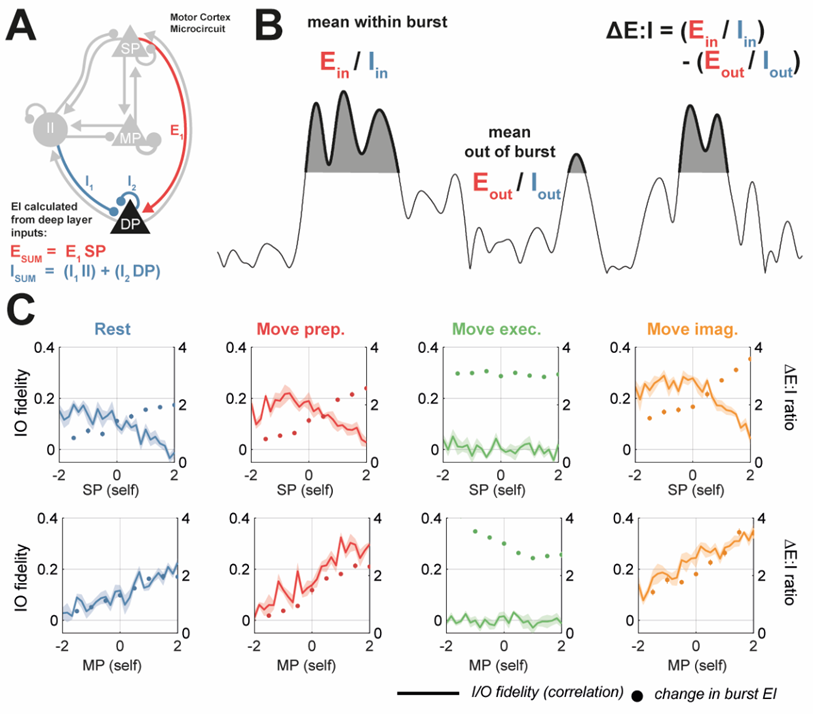


**Supplementary Figure 6 – Analysis of the relationship between changes in Excitation/Inhibition ratios (EI) during bursts and the relationship with cortical input transmission. (A)** The EI balance was calculated as the ratio in the sums of excitatory and inhibitory synaptic inputs to the deep cell layers, the main output of the motor cortex. **(B)** The change in the EI balance when in and out of bursts was computed from simulated activity in the spontaneous model. **(C)** The strength of layer specific self-inhibition in either superficial (SP self; top row) or middle (MP self; bottom row) layers was modulated. The change in within burst EI ratio was then plot (right axis; circles) against the input/output fidelity (left axis; bold lines).

## Supplementary Figure 7


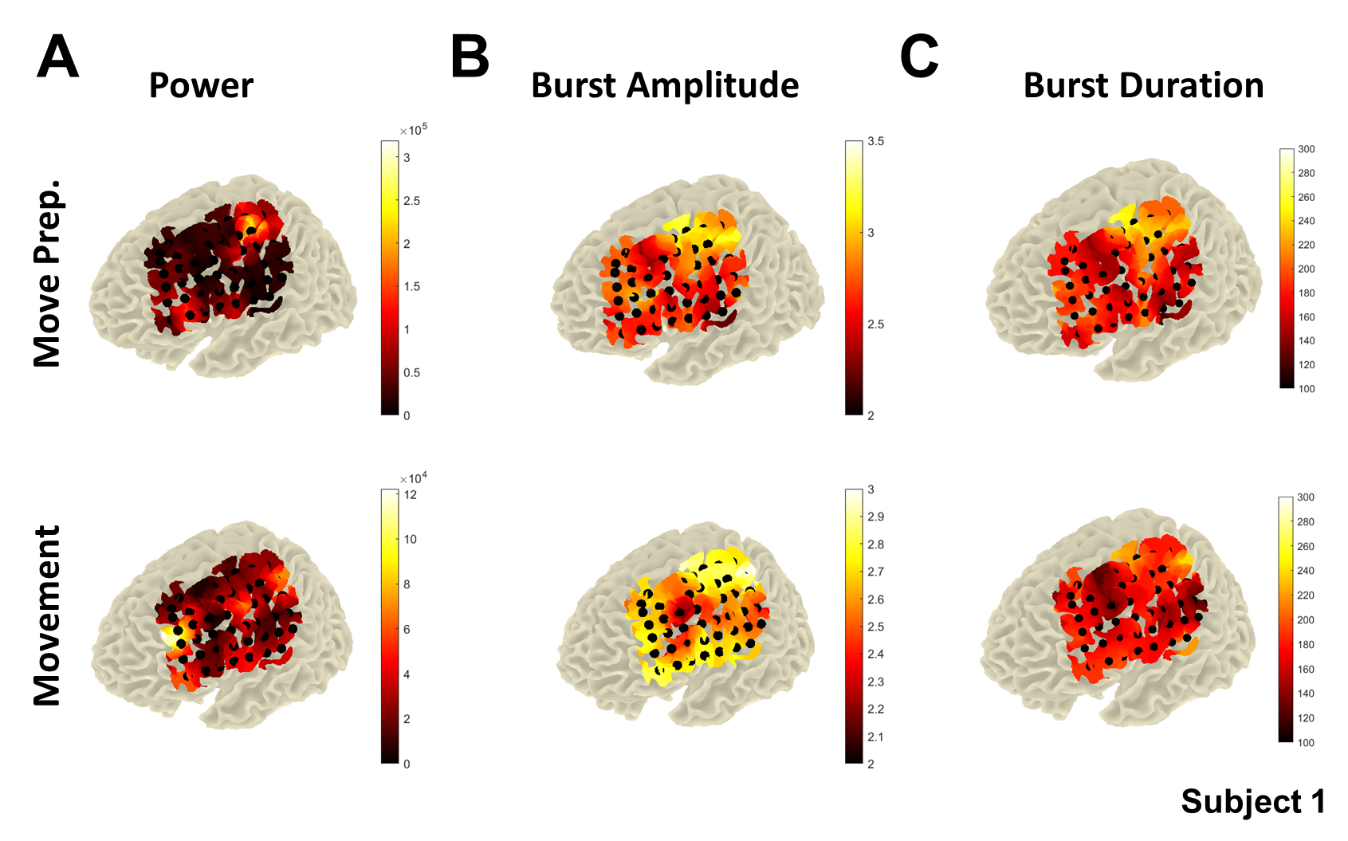


**Supplementary Figure 7 – Example spatial distributions of spectral power, mean burst amplitude and burst duration at the beta frequency (14-30 Hz) from subject 1.** Black dots indicate approximate ECoG electrode locations. Functional data was projected onto the cortical surface using spherical interpolation weighted by the distance from the sensors. **(A)** Distribution of average beta power across the cortical surface. **(B)** Distribution of average burst amplitude (Z-scored). **(C)** Distribution of average burst duration (ms).

## Supplementary Figure 8


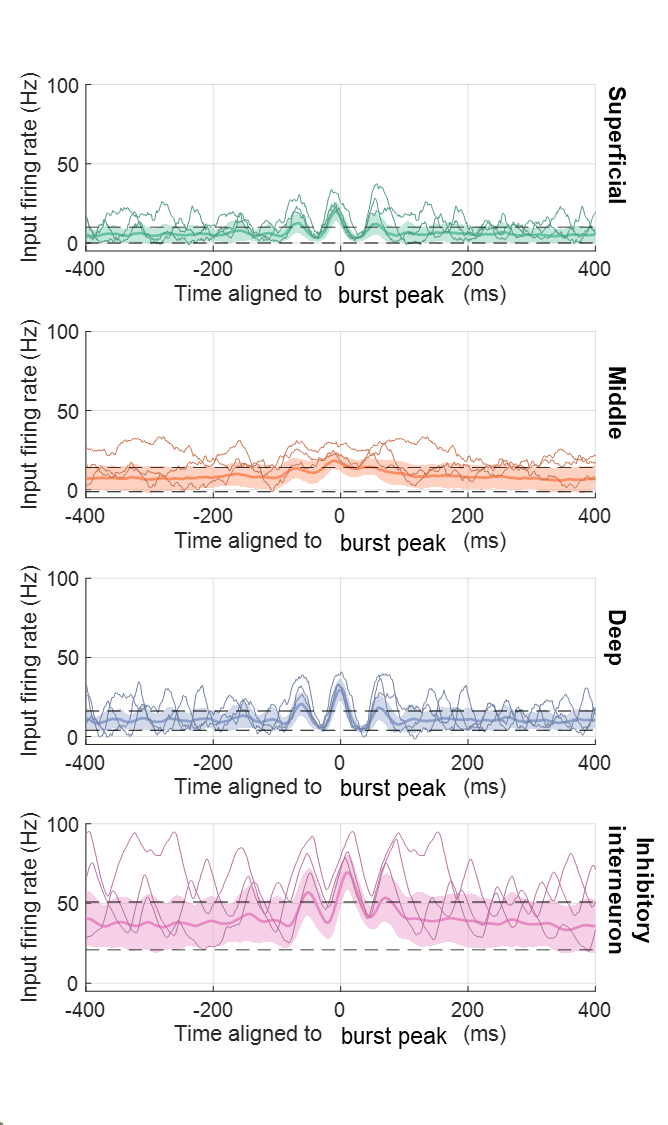


**Supplementary Figure 8- Analysis of simulated model activity to show laminar specific activations during high amplitude beta bursts.** The Wilson-Cowan model of the motor microcircuit, with parameters fit to the group averaged spectra and burst features of the movement preparation data was simulated for 48s. Bursts were detected as detailed in supplementary methods III. Bursts were aligned to the peak of the band-passed filtered signal. The bold line and shaded bounds indicate the mean and standard deviation of the bursts in the unfiltered firing rates of each layer. The dashed lines give the standard deviation of the firing rates across the whole trace. The top three highest amplitude bursts are overlaid.

## Supplementary Methods

### Supplementary Methods I –Wide/Narrow-band SNR Calculations

The signal-to-noise (SNR) characteristics of signals were determined by the following equation:

$$SNR_{dB}=10\log_{10} \left( \frac{P_{signal}}{P_{noise}} \right)$$

Equation 1.

In the case of narrow-band calculations (intended to determine the magnitude of the beta peak with respect to the underlying noise floor), $P_{signal}$ is given by the average power at the peak frequency ±3 Hz, with the peak detected within the range 12-30 Hz. $P_{noise}$ is given by the average power in the tail of the spectrum at 48-98 Hz.

Wide-band SNR was computed by taking $P_{signal}$ to be equal to the average power in the range 4-48 Hz, $P_{noise}$as the average of the power in the amplifier noise floor in the same frequency range.

### Supplementary Methods II – Spectral Reduction

Spectra were preprocessed prior to ABC model fitting in order to remove the aperiodic 1/f background such that fits were focussed on beta band activity. Peaks in the power spectrum in the beta frequency range were found using the *findpeaks* algorithm implemented in MATLAB. Prior to peak finding, spectra were smoothed with a 5 Hz wide Gaussian kernel. Inflection points (i.e., the troughs separating peaks) were then determined by finding the nearest sign change of the approximate derivative (difference) from each peak. This then defined the frequency range over which a Cauchy function was fit. This procedure was formed for each peak. The composite spectra were then formed from the non-overlapping sum of each fitted model.

### Supplementary Methods III – Definition of Bursts

Bursts were defined by setting a threshold on the bandlimited envelope. The filter passband was set at ±5 Hz of the peak frequency and implemented using a zero-phase FIR filter. Filtered data were then Z-normalised. The analytic signal was constructed using the Hilbert transform to estimate instantaneous amplitude. Bursts were defined as periods exceeding the 75^th^ percentile of this envelope and the minimum burst length was set to two periods of the upper limit of the bandpass filter unique to each dataset. Bursts found at the boundaries of epochs were discarded from the analysis. Burst amplitudes were taken as the maximum of the envelope within each burst, whilst burst duration reflects the amount of time that the envelope exceeds the threshold. Inter-burst intervals represent the time spent sub-threshold between each event. To summarise burst features, we estimated distributions of burst duration, amplitude, and inter-burst intervals using binned histograms. Distributions were then estimated using a kernel density estimate of the probability density function specifying a standard normal function for the kernel. Please note that because we set a minimum burst duration, the true underlying distribution is truncated. For a given probability density *p(x)* with support *x*, the mean and standard deviation were computed as the expected value and standard deviation using: $\mu=\int x p\left( x \right)dx$ and $\sigma^{2}=\int\left( x-\mu\right)^{2} p\left( x \right) dx$. For brevity we refer to this expected value as the “mean” inside of the main text.

### Supplementary Methods IV –Model Formulation

The model uses the firing rate equations (Vogels et al. 2005; Wilson and Cowan 1972) constructed with the same architecture outlined in (Bhatt et al. 2016). The average firing rate of each laminar population (middle *MP*, superficial *SP*, inhibitory interneuron *II*, deep *DP*) is given by the following state equations:

$$\frac{dR_{MP}}{dt}=\frac{1}{T_{MP}}\left( -R_{MP}+S\left( \left\{ {-G}_{MP\to MP}R_{MP}-G_{II\to MP}R_{II}+ G_{SP\to MP}R_{SP} \right\},M_{MP},S_{MP},B_{MP} \right) \right)$$

$$\frac{dR_{SP}}{dt}=\frac{1}{T_{SP}}\left( -R_{SP}+S\left( \left\{ {-G}_{SP\to SP}R_{SP}+G_{MP\to SP}R_{MP}-G_{II\to SP}R_{II}+G_{DP\to SP}R_{DP} \right\},M_{SP},S_{SP},B_{SP} \right) \right)$$

$$\frac{dR_{II}}{dt}=\frac{1}{T_{II}}\left( -R_{II}+S\left( \left\{ -G_{II\to II}R_{II}+G_{MP\to II}R_{MP}+G_{DP\to II}R_{DP}+G_{SP\to II}R_{SP} \right\},M_{II},S_{II},B_{II} \right) \right)$$

$$\frac{dR_{DP}}{dt}=\frac{1}{T_{DP}}\left( -R_{DP}+S\left( \left\{ {-G}_{DP\to DP}R_{DP}-G_{II\to DP}R_{II}+G_{SP\to DP}R_{SP} \right\},M_{DP},S_{DP},B_{DP} \right) \right)$$

Equation 2.

Where *T* gives the population time constant, *G* gives the weight of the (delayed) synaptic connection, and *S(I,M,S,B)* reflects the sigmoidal transfer function for the total input *I* given within the curly braces:

$$S\left( I,M,S,B \right)=\frac{M}{1+{exp}^{\frac{-S I}{M}}\cdot\frac{(M-B)}{B}}$$

Equation 3.

where *M* reflects the maximum firing rate, *S* the slope of the sigmoid, and *B* the spontaneous firing rate (i.e., baseline firing rate in the absence of input). Many of the values of these parameters can be ascertained from empirical estimates available from online databases (see supplementary table I). The model includes finite transmission delays using delayed values of *R*, i.e., the delayed input from the *j^th^* to the *i^th^* population is given by:

$I_{j\to i}(t)=G_{j\to i}R_{j}(t-\tau_{j\to i})$)

Equation 4.

where $\tau_{j\to i}$ reflects the finite time delay. Each state receives stochastic innovations added to the deterministic equations (given above). Delays were discretized and rounded to the nearest integration step size. Stochastic inputs were given by rescaling the variance of the noise to match the square root of the integration step *h* (i.e., $dW_{t}=W_{t+h}-W_{t}\sim N\left( 0,h \right)$, where $W_{t}$ is a Wiener process, and $N$ refers to the normal distribution. The system of equations was then integrated using an Euler-Maruyama scheme with fixed step size of 0.5 ms.

## Supplementary Methods V- Bayesian Model Comparison

Model comparison with ABC was performed using a marginal likelihood based approach (Toni et al. 2009). The marginal likelihood (model evidence) for each *j^th^* model:

$$P(D_{0}|M^{j})\cong\frac{\# \left\{ \rho\left( \mu_{n},\mu_{0} \right)\leq\hat{\epsilon} \right\}}{N}$$

Equation 5.

where $\hat{\epsilon}$ is a threshold on the distance metric $\rho$. We set $\epsilon^{*}$ to median distance of the best fitting model, using R^2^ as a distance metric. The marginal posterior probability of a model is then given by combining marginal model likelihoods and prior model probabilities, and then normalizing across the full model space. For more details of the procedure please see West et al. (2021).

## Supplementary Methods VI- Projection of Functional ECoG Data onto the Cortical Surface

Electrode positions were localized from a combination of preoperative MRI and postoperative CT scans using procedures detailed in Miller (2019). These localizations as well as a cortical mesh, segmented from imaging, are precomputed, and provided in the data repository from Miller. Functional data features were computed for each electrode (as detailed in supplementary methods I and III). This data was then projected onto the cortical surface using ‘ft_sourceplot’ (Stolk et al. 2018).

## Supplementary Methods VII – Jansen-Rit Neural Mass Model

As detailed in supplementary methods V, we also included a Jansen-Rit formulation (Jansen and Rit 1995) of the motor cortex microcircuit (Bhatt et al. 2016) in our initial Bayesian model comparison. The dynamics described by the Jansen-Rit model are given by:

$\dot{v_{i}}=x_{i}$,

$$\dot{x_{i}}=\frac{1}{\tau_{i}}\left( {A_{ij}S}_{i}\left( v_{j} \right)+u_{i} \right)-\frac{2}{\tau_{i}}x_{i}-\frac{1}{\tau_{i}^{2}}v_{i},$$

Equation 6.

where the average postsynaptic membrane potential of the *i^th^* population is given by $v_{i}$, with a time scale set by the lumped post synaptic time constant $\tau_{i}$. The input to the mass is given between the outer set of brackets and comprises some background noise $u_{i}$ plus an input from projecting population *j* passed through a sigmoidal transfer function *S_i_* and weighted by the connection strength *A_ij_*_._ Nodes were coupled with the same architecture described for the Wilson-Cowan model (section 7.3.4). For a full description of the motor cortex circuit, please see (West et al. 2022).

## Supplementary Methods VIII – Full ethics statements

The following ethics statements appear in their original, unmodified state as given in the terms of sharing of the data repository.

**Cued Finger Movements**

“All patients participated in a purely voluntary manner, after providing informed written consent, under experimental protocols approved by the Institutional Review Board of the University of Washington (#12193). All patient data was anonymized according to IRB protocol, in accordance with HIPAA mandate. These data originally appeared in the manuscript “Human Motor Cortical Activity Is Selectively Phase- Entrained on Underlying Rhythms” published in PLoS Computational Biology in 2012 (Miller et al. 2012).”

**Movement Imagery**

“All patients participated in a purely voluntary manner, after providing informed written consent, under experimental protocols approved by the Institutional Review Board of the University of Washington (#12193). Portions of these data originally appeared in the manuscript “Cortical activity during motor execution, motor imagery, and imagery-based online feedback” published in PNAS in 2010 (Miller et al. 2010). Portions of these patient data was anonymized according to IRB protocol, in accordance with HIPAA mandate. It was made available through the library described in “A Library of Human Electrocorticographic Data and Analyses” by Kai Miller (Miller 2019), freely available at <https://searchworks.stanford.edu/view/zk881ps0522>.”

**Basic Motor**

“Ethics statement: All patients participated in a purely voluntary manner, after providing informed written consent, under experimental protocols approved by the Institutional Review Board of the University of Washington (#12193). All patient data was anonymized according to IRB protocol, in accordance with HIPAA mandate. It was made available through the library described in “A Library of Human Electrocorticographic Data and Analyses” by Kai Miller (Miller 2019), freely available at https://searchworks.stanford.edu/view/zk881ps0522. All patient data was anonymized according to IRB protocol, in accordance with HIPAA mandate. These data originally appeared in the manuscript “Spectral Changes in Cortical Surface Potentials during Motor Movement” published in Journal of Neuroscience in 2007 (Miller et al. 2007).”

### Supplementary Table I – Prior Model Parameters

Where possible we derived prior estimates from empirical sources available from either the Allen Brain Atlas, or Neuroelectro.org. Estimates derived from human cells were preferred, but when not available, estimates in animals were also used. Estimates of prior precision (i.e., inverse variance) were obtained by looking at the variance in independently reported measurements.

| Parameter | mean | variance | units | reference(s) |
| --- | --- | --- | --- | --- |
| Synaptic weights |  |  |  |  |
| SP → II | 12 | 0.25 | mv.s |  |
| SP → MP | 12 | 0.25 | mv.s |  |
| SP → DP | 6 | 0.25 | mv.s |  |
| SP → SP | 12 | 0.25 | mv.s |  |
| II → SP | 12 | 0.25 | mv.s |  |
| MP → MP | 12 | 0.25 | mv.s |  |
| MP → SP | 12 | 0.25 | mv.s |  |
| DP → SP | 6 | 0.25 | mv.s |  |
| DP → II | 6 | 0.25 | mv.s |  |
| DP → DP | 3 | 0.25 | mv.s |  |
| II → DP | 6 | 0.25 | mv.s |  |
|  |  |  |  |  |
| Transmission delays |  |  |  |  |
| MP → MP | 0.001 | 0.0005 | s |  |
| MP → SP | 0.002 | 0.0005 | s |  |
| II → MP | 0.002 | 0.0005 | s |  |
| II → II | 0.001 | 0.0005 | s |  |
| MP → II | 0.002 | 0.0005 | s |  |
| DP → II | 0.002 | 0.0005 | s |  |
| SP → SP | 0.001 | 0.0005 | s |  |
| SP → MP | 0.002 | 0.0005 | s |  |
| II → DP | 0.002 | 0.0005 | s |  |
| DP → DP | 0.001 | 0.0005 | s |  |
| SP → DP | 0.003 | 0.0005 | s |  |
| II → SP | 0.002 | 0.0005 | s |  |
| SP → II | 0.002 | 0.0005 | s |  |
| DP → SP | 0.003 | 0.0005 | s |  |
|  |  |  |  |  |
| Time constants |  |  |  |  |
| τ_mp_ | 0.025 | 0.020 | s | Allen Cell Atlas:  L4 Spiny Human |
| τ_sp_ | 0.020 | 0.012 | s | Allen Cell Atlas:  L2 Spiny Human |
| τ_ii_ | 0.015 | 0.006 | s | Allen Cell Atlas:  L2/3/4/5 Aspiny Human |
| τ_dp_ | 0.015 | 0.015 | s | Allen Cell Atlas:  L5/6 Spiny Human |
|  |  |  |  |  |
| Input gain |  |  |  |  |
| C_mp_ | 20 |  | sp.s^-1^ |  |
| C_sp_ | 20 |  | sp.s^-1^ |  |
| C_ii_ | 20 |  | sp.s^-1^ |  |
| C_dp_ | 20 |  | sp.s^-1^ |  |
|  |  |  |  |  |
| Maximum firing rates |  |  |  | Taken from Neuroelectro.org |
| Mn_mp_ | 67 | 11 | sp.s^-1^ |  |
| Mn_sp_ | 64 | 55 | sp.s^-1^ |  |
| Mn_ii_ | 131 | 106 | sp.s^-1^ |  |
| Mn_dp_ | 44 | 17 | sp.s^-1^ |  |
|  |  |  |  |  |
| Slope of the sigmoid |  |  |  |  |
| Sn_mp_ | 0.1 | 0.1 | sp.s^-1^.pA^-1^ | Allen Cell Atlas:  L4 Spiny Human |
| Sn_sp_ | 0.2 | 0.2 | sp.s^-1^.pA^-1^ | Allen Cell Atlas:  L2/3 Spiny Human |
| Sn_ii_ | 0.4 | 0.2 | sp.s^-1^.pA^-1^ | Allen Cell Atlas: L2/3/4/5 Aspiny Human |
| Sn_sp_ | 0.1 | 0.15 | sp.s^-1^.pA^-1^ | Allen Cell Atlas:  L5/6 Spiny Human |
|  |  |  |  |  |
| Basal firing rates |  |  |  | Taken from Neuroelectro.org |
| Bn_mp_ | 18 | 5 | sp.s^-1^ |  |
| Bn_sp_ | 5 | 5 | sp.s^-1^ |  |
| Bn_ii_ | 20 | 20 | sp.s^-1^ |  |
| Bn_dp_ | 10 | 5 | sp.s^-1^ |  |
|  |  |  |  |  |
| Observation noise gain |  |  |  |  |
| C_obs_ | 0.2 |  | Scalar |  |
|  |  |  |  |  |
|  |  |  |  |  |
| Leadfield |  |  |  |  |
| L_obs_ | [0.1 0.3 0.1 0.5] |  |  |  |

## Table of External Software

We thank the authors of the 3^rd^ party software used in this work and acknowledge them below.

| Toolbox Name | Author | Year | Source/Reference |
| --- | --- | --- | --- |
| boundedline-pkg | Kelly Kearney | 2015 | <https://github.com/kakearney/boundedline-pkg> |
| brewermap | Stephen Cobeldick | 2014 | <https://github.com/DrosteEffect/BrewerMap> |
| colouredNoise | B. Scott Jackson; John Burkardt | 2005-2010 |  |
| drtoolbox | Laurens van der Maaten |  | <https://lvdmaaten.github.io/drtoolbox/> |
| fdr_bh | David Groppe | 2015 | <https://uk.mathworks.com/matlabcentral/fileexchange/27418-fdr_bh> |
| Fieldtrip | Donders Institute, Radbound University | 2020 | <https://www.fieldtriptoolbox.org/> |
| linspecer | Jonathan C. Lansey | 2015 | <https://github.com/davidkun/linspecer> |
| neurospec 2.2 | David Halliday | 2018 | [https://www.neurospec.org/](http://www.neurospec.org/) |
| splitvec | Bruno Luong | 2009 | <https://uk.mathworks.com/matlabcentral/fileexchange/24255-splitvec> |
| SPM 12 | Wellcome Centre for Human Neuroimaging, University College London | 2020 | <https://www.fil.ion.ucl.ac.uk/spm/> |
